# Phage-Encoded Sigma Factors Alter Bacterial Dormancy

**DOI:** 10.1128/msphere.00297-22

**Published:** 2022-07-20

**Authors:** D. A. Schwartz, B. K. Lehmkuhl, J. T. Lennon

**Affiliations:** a Department of Biology, Indiana University, Bloomington, Indiana, USA; University of Michigan—Ann Arbor

**Keywords:** *Bacillus*, auxiliary metabolic genes, dormancy, evolution, infection, parasite, phage, seed banks, sporulation, starvation, transcriptome, virus

## Abstract

By entering a reversible state of reduced metabolic activity, dormant microorganisms are able to tolerate suboptimal conditions that would otherwise reduce their fitness. Dormancy may also benefit bacteria by serving as a refuge from parasitic infections. Here, we focus on dormancy in the *Bacillota*, where endospore development is transcriptionally regulated by the expression of sigma factors. A disruption of this process could influence the survivorship or reproduction of phages that infect spore-forming hosts with implications for coevolutionary dynamics. We characterized the distribution of sigma factors in over 4,000 genomes of diverse phages capable of infecting hosts that span the bacterial domain. From this, we identified homologs of sporulation-specific sigma factors in phages that infect spore-forming hosts. Unlike sigma factors required for phage reproduction, we provide evidence that sporulation-like sigma factors are nonessential for lytic infection. However, when expressed in the spore-forming Bacillus subtilis, some of these phage-derived sigma factors can activate the bacterial sporulation gene network and lead to a reduction in spore yield. Our findings suggest that the acquisition of host-like transcriptional regulators may allow phages to manipulate a complex and ancient trait in one of the most abundant cell types on Earth.

**IMPORTANCE** As obligate parasites, phages exert strong top-down pressure on host populations with eco-evolutionary implications for community dynamics and ecosystem functioning. The process of phage infection, however, is constrained by bottom-up processes that influence the energetic and nutritional status of susceptible hosts. Many phages have acquired auxiliary genes from bacteria, which can be used to exploit host metabolism with consequences for phage fitness. In this study, we demonstrate that phages infecting spore-forming bacteria carry homologs of sigma factors, which their hosts use to orchestrate gene expression during spore development. By tapping into regulatory gene networks, phages may manipulate the physiology and survival strategies of nongrowing bacteria in ways that influence host-parasite coevolution.

## INTRODUCTION

Dormancy is a life history strategy that allows individuals to enter a reversible state of reduced metabolic activity. An example of convergent evolution, it has independently arisen throughout the tree of life as a means of coping with fluctuating and unpredictable environments (1). Dormancy is particularly prevalent among microbial life forms where it contributes to the persistence and fitness of populations in environments where variables such as pH, oxygen, and resource availability are suboptimal for growth and reproduction (2). In addition to buffering populations against abiotic features of the environment, dormancy may be reinforced through dynamics that arise from species interactions. For example, dormancy diminishes the strength of competition, which in turn can promote species coexistence (3). In addition, dormancy may benefit populations by serving as a refuge against predator consumption or parasite infection (4, 5).

Among microorganisms, dormancy can protect hosts from viral parasites in a number of ways. As cells transition into an inactive state, they often undergo morphological changes that affect how viruses physically interact with their host. The formation of dormant cells, such as cysts and spores, often involves the development of a thick exterior coating (6–8) that masks the surface molecules used by viruses for attachment (9–12). Even if a virus is able to gain entry into a dormant cell, parasite productivity will be low owing to constraints imposed by the host’s reduced metabolism (13–16). Furthermore, viral defense genes are often located in proximity to genes that regulate dormancy and cell suicide, suggesting that dormancy may contribute to multilayered protection against viral infection (17, 18). For example, virus-induced dormancy has been linked to CRISPR-Cas systems in bacteria and archaea (19, 20). As a physiological refuge (21), dormancy can confer herd immunity and diminish the spread of epidemics (22), which may ultimately shape host-virus coevolutionary dynamics.

A take-home lesson from studies on antagonistic coevolution is that host defenses are prone to being overcome by viruses (23, 24). One general mode of virus adaptation involves the acquisition of host genes. Viral genomes commonly encode homologs of genes that are involved in host metabolism. These so-called auxiliary genes can alter cellular processes in ways that affect virus fitness (25). Originally motivated by the discovery of photosynthesis genes in marine cyanophages (26, 27), auxiliary genes have been implicated in host nutrition (nitrogen and phosphorus metabolism) and energy acquisition (sulfur oxidation and fermentation), along with basic cellular functions such as protein translation and bacterial communication via quorum sensing (28–30). In addition, some virus genomes contain host defense genes, which has led to speculation that auxiliary genes may modify parasite infectivity and reproduction (31, 32). Similarly, some phage genomes have been reported to have genes similar to those required for the development of resting structures, such as spores that are critical for certain types of dormancy (33–42). Phages might use sporulation homologs to inhibit their host from entering a dormancy refuge, thereby enhancing the reproductive component of parasite fitness. Alternatively, phages might exploit sporulation in a way that extends longevity and thereby enhance the survivorship component of fitness. This could happen through a process known as entrapment whereby a phage genome is translocated into the developing spore resulting in the production of a “virospore” (12, 43–46). Analogous to pseudolysogeny, the phage genome is protected by the endospore from conditions that would otherwise contribute to phage decay without it being integrated into the host chromosome (45). When environmental conditions improve, the dormant cell undergoes germination, and the phage resumes its lytic reproductive cycle. For example, when phage phi29 infects Bacillus subtilis, it prioritizes entrapment by repressing lytic activity in vegetative cells that are in the process of undergoing sporulation (47).

As a complex form of dormancy, sporulation presents phages with many opportunities for intervention. For proper development, sporulation requires the coordinated regulation of a large gene network (48–50). The central regulatory module of sporulation relies on the activity of sigma factors, the exchangeable subunit of the transcriptional machinery that dictates promoter specificity of RNA polymerase (51). Among bacteria, a primary sigma factor is responsible for regulating growth, reproduction, and other housekeeping processes (52). Under conditions that are unfavorable for growth, the primary sigma factor is replaced by alternative sigma factors. For example, during B. subtilis spore development, *sigA* is swapped out by a cascade of sporulation-specific sigma factors, each driving the expression of a subset of sporulation genes in distinct cellular compartments at specific times (53). Following an asymmetrical cell division, gene expression in the maturing spore (i.e., forespore) is driven first by *sigF* and then *sigG*, while expression in the mother cell is driven by *sigE* and then *sigK*. Because alternative sigma factors are only transiently used, their deletion is typically nonlethal, and thus they are considered nonessential.

Like sporulation, phage development relies on synchronized gene expression, which can be accomplished in a variety of ways. For example, they can use host-encoded sigma factors, encode sigma factor analogs (e.g., gp33 in T4), or use their own RNA polymerases that do not require sigma factors (51, 54–56). In addition, some phages encode their own sigma factors. In fact, early investigations of phage-encoded sigma factors helped elucidate how the swapping of sigma subunits controls gene transcription by RNA polymerase (51). However, phage-encoded sigma factors regulating lytic development are quite divergent from bacterially encoded sigma factors (56, 57). More recently, genomic data have identified phage-encoded sigma factors that bear a greater resemblance to bacterial sigma factors, especially those involved in the regulation of sporulation in the *Bacillota*, the phylum of bacteria formerly known as the *Firmicutes* (34, 41, 42, 58). While the function of these homologs for phages remains unknown, previous experimental studies have suggested that sporulation-like sigma factors found in several B. anthracis phages retain a function that is relevant to host sporulation (41, 42, 58). Therefore, it is possible that sporulation-like sigma factors have been coopted by phages to manipulate host dormancy in ways that could potentially enhance their reproduction or survival.

Here, we use a combination of bioinformatics and laboratory experiments to test whether homologs of sporulation-specific sigma factors can be used by phages to manipulate host dormancy. Using sequence homology and phylogenetic analyses, we identify and classify hundreds of phage-encoded sigma factors. We find that phages capable of infecting spore-forming hosts preferentially encode sigma factors that are homologous to the forespore-specific sigma factors, *sigF* and *sigG*. When homologs of sporulation-specific sigma factors are expressed in B. subtilis, we observed that conserved phage-encoded homologs alter host gene expression and reduce spore yield. Together, our findings have implications for understanding dormancy dynamics in environmental, engineered, and host-associated ecosystems where endospores constitute one of the most abundant cell types on Earth (59).

## RESULTS

### Sporulation-like sigma factors recovered from phages that infect spore-forming hosts.

**FIG 1:**
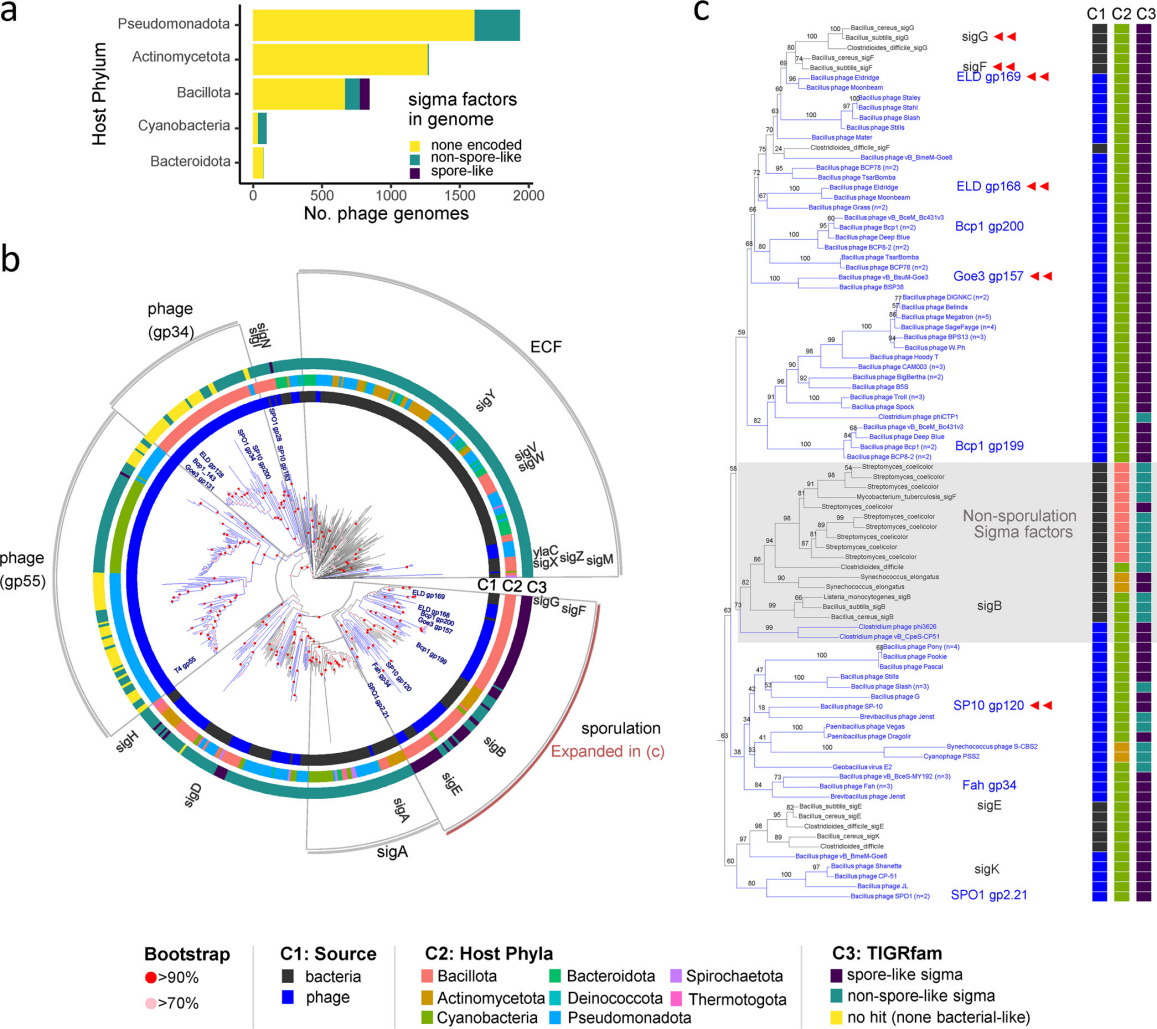
Distribution and classification of phage-encoded sigma factors. (a) Distribution of sigma factors classes in phage genomes by host phyla. Sporulation-like sigma factors from the viral orthologous groups (VOG) database were classified using phylogeny and TIGRFAM homology as either sporulation-like (“spore-like”) or not (“non-spore-like”). A phage genome that contains both spore-like and non-spore-like sigma factors is marked as spore-like. Host phyla with <10 phage genomes are not shown. (b) Phylogenetic tree representing proteins of phage-encoded sigma factors from VOG. For reference, we included sigma factors from 24 genomes of diverse bacterial species. Blue branches on the tree represent monophyletic clades that only contain phage-encoded sigma factors. Black branches represent bacterium-encoded sigma factors and the internal branches leading to them. Phage proteins discussed in the main text are labeled at branch tips (e.g., ELD gp128). In circle 1 (C1), we identify the source (phage- versus bacterium-encoded) of a sigma factor protein. In circle 2 (C2), we designate the taxonomy of the bacterial host (phyla). In circle 3 (C3), we provide the best hit (hmmscan sequence E value) of the homolog to bacterium-encoded sigma factors families in TIGRfam. Outside of circle 3, we indicate the relative positions of Bacillus subtilis sigma factor genes and depict the different sigma factor families (gray wedges). Branch nodes are labeled with nonparametric bootstrap support values (*n* = 1,000). (c) Clade of sporulation-specific sigma factors depicted by a red arc in panel b. Within this clade, the subclade of *sigB* and its relatives (gray background) are not associated with sporulation.

To characterize the distribution of sporulation-like sigma factors encoded by phages, we classified all proteins defined as sigma factors (*n* = 686) in the database of viral orthologous groups (VOG). Phage genes in this database come from viral RefSeq genomes, most of which were sequenced from isolates with known hosts. The majority (86.6%) of phage genomes in the VOG database (*n* = 4,350) lack sigma factors (Fig. 1a). The remaining genomes contain one (11.7%), two (1.1%), or three (0.7%) sigma-factor homologs. Among these, we identified homologs of bacterial sporulation-like sigma factors using two classification methods (Fig. 1). First, for each phage-encoded sigma factor, we determined homology to bacterial sigma factor protein families (TIGRFAMs) using hidden Markov models (HMMs). Second, we constructed a phylogeny of the VOG sigma factors together with sigma factors from diverse bacteria and identified phage genes that clustered within the group of bacterial sporulation-specific sigma factors (Fig. 1c). Results from the two approaches were in high agreement. Of the 89 homologs identified by phylogeny and the 94 homologs identified by HMMs, there were 82 sigma factors that were classified as sporulation-like by both methods.

These classified sporulation-like sigma factors were only found in phages capable of infecting *Bacillota*, the bacterial phylum that contains endospore-forming bacteria (Fig. 1a). The sporulation-like sigma factors were further restricted to phages that infect spore-forming genera with the *Bacillota* (see Fig. S1 in the supplemental material). In contrast, sporulation-like sigma factors were found in a diverse set of phages belonging to three viral families (*Herelleviridae*, *Siphoviridae*, and *Myoviridae*; see Fig. S1 in the supplemental material). Furthermore, sporulation-like sigma factors have been identified in temperate phages (WBeta [42], vB_BceS-MY192 [60], and PfEFR-4 and PfEFR-5 [61]), as well as in strictly lytic phages (e.g., SPO1 and other members of the *Herelleviridae* [36, 62]), suggesting that that acquisition of these genes is not restricted to a particular viral life-history strategy.

10.1128/msphere.00297-22.1 FIG S1 Distribution of sigma factors in *Bacillota* phages. Each colored rectangle represents a single sigma factor gene encoded in the genome of the phage whose name is indicated in the row label. Panels separate phages by viral family (panel titles in black), and the host genera are depicted in the upper right of each subpanel. Text color of the genus name corresponds to endospore-forming (blue) versus non-endospore-forming (red) genera, as defined by *Bergey’s Manual of Systematic Bacteriology* (volume 3, The *Firmicutes*, 2011; Springer Science & Business Media). Download FIG S1, PDF file, 0.4 MB. Copyright © 2022 Schwartz et al. 2022 Schwartz et al. https://creativecommons.org/licenses/by/4.0/ This content is distributed under the terms of the Creative Commons Attribution 4.0 International license.

### A sporulation-like sigma factor is nonessential for phage reproduction.

**FIG 2:**
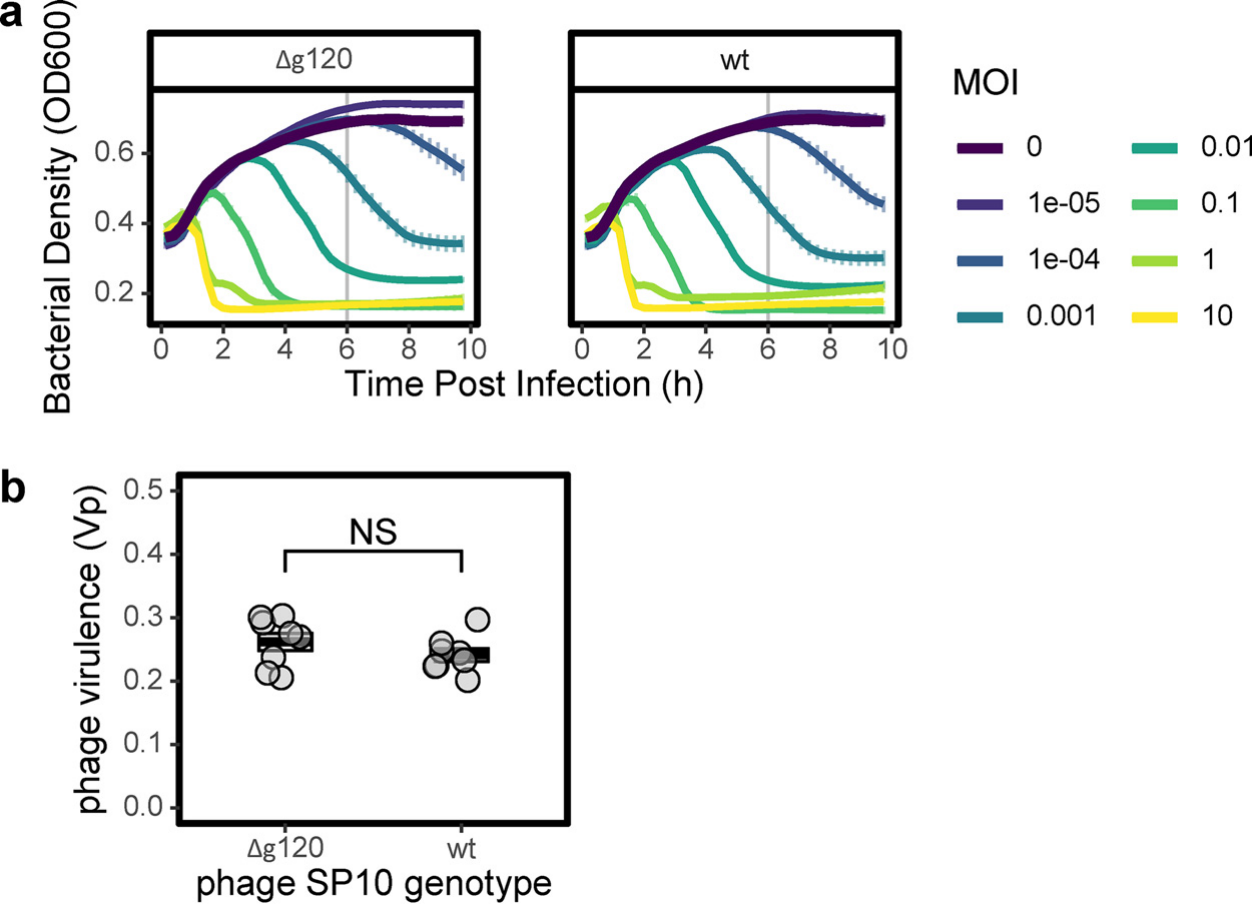
A sporulation-specific sigma factor is nonessential for phage virulence. Phage SP10 from which we deleted the sporulation sigma factor homolog g120 (Δg120) is equally virulent to the wild-type (WT) phage when infecting Bacillus subtilis Δ6. (a) To quantify phage virulence (Vp), we measured the optical density (OD_600_, means ± the standard errors of the mean [SEM], *n* = 8) over a range of multiplicities of infection (MOI = phage/bacterium titers). To calculate the Vp, we integrated over 6 h following infection (vertical gray line), which corresponded to the time the bacterial population transitioned from the exponential growth phase to the stationary phase in noninfected cultures (MOI = 0). (b) There was no difference in virulence between phage with versus without the sporulation-like sigma factor (*t*_12.9_ = 1.22, *P = *0.25; *n* = 8).

Despite similarities to the host genes, it is possible that phage-encoded sigma factors could be used by phages to regulate essential functions that are unrelated to sporulation, such as genome replication and capsid assembly. To test this hypothesis, we set out to delete the coding sequences of sporulation-like sigma factors from phage genomes. If these mutants retained infectivity, it would suggest that the sporulation homologs were nonessential and that they perhaps have auxiliary function (25, 63). Using a CRISPR-Cas9 editing system (64), we attempted to delete g120 from SP10 (65) and g157 from Goe3 (66), which are both phages that infect B. subtilis. Despite multiple attempts, we were unable to construct the plasmid required to delete g157 from phage Goe3. This could potentially reflect toxicity of the flanking regions of the target gene toward the *Escherichia coli* cloning strain (67). However, we were able to delete the entire coding sequence of g120 from the genome of phage SP10. The resulting mutant could still productively infect its host, demonstrating that the sporulation-like homolog was nonessential for phage reproduction under standard laboratory conditions. In fact, there was no detectable reduction of virulence when infecting B. subtilis with the mutant phage (*t*_12.9_ = 1.22, *P = *0.25; Fig. 2).

### Phage-encoded sigma factors alter expression of host sporulation genes.

**FIG 3:**
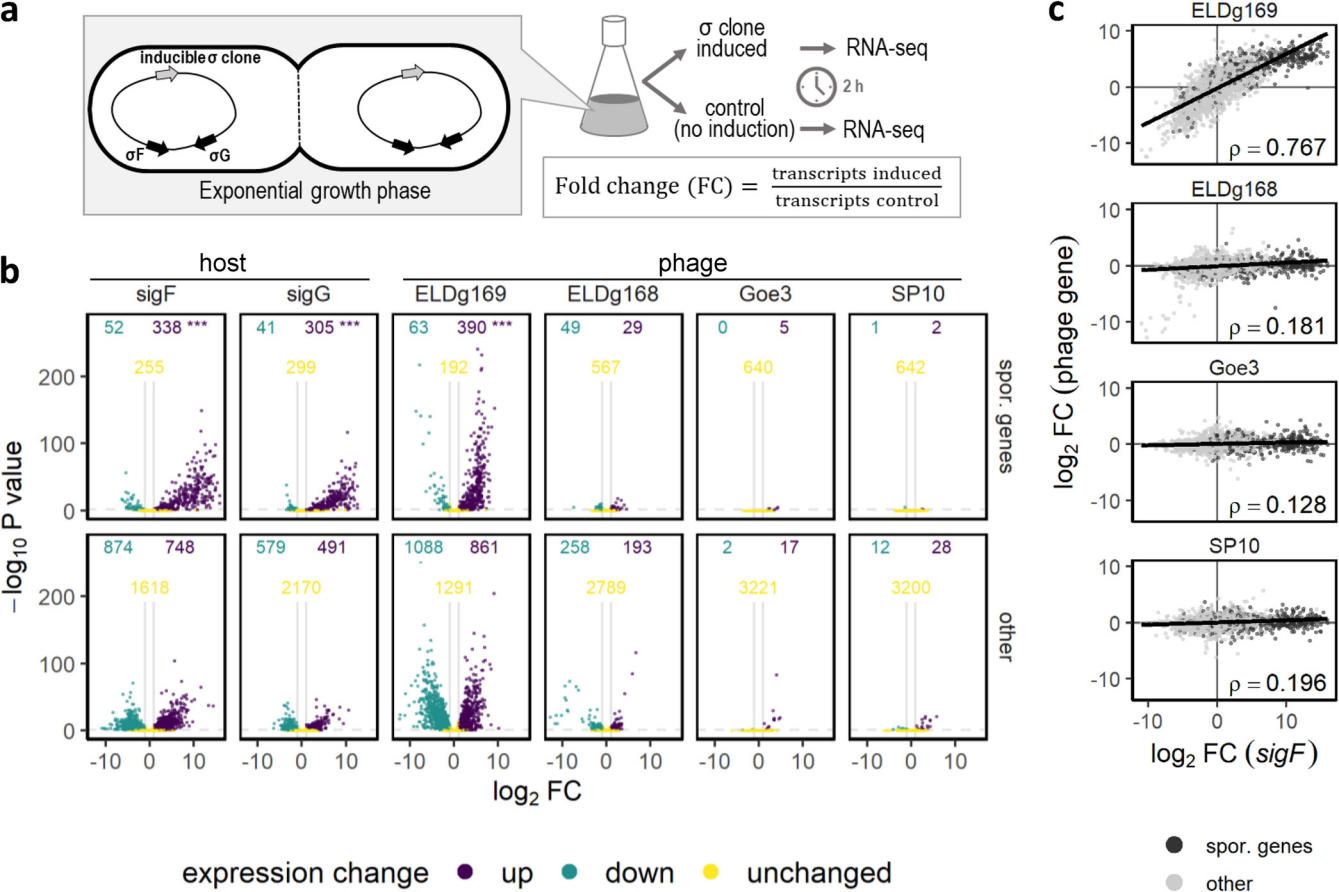
Bacterial gene expression following induction of phage-derived sigma factors. (a) Illustration of the experimental design. Phage- and host-derived sigma factors were cloned into Bacillus subtilis under an IPTG-inducible promoter (gray arrow) in a strain (Δ6) that contains a fully functional sporulation gene network, including its native sporulation-specific sigma factors (black arrows depict representative examples). To estimate differential expression, we compared splits of B. subtilis cultures. One half was induced to express a cloned sigma factor during exponential growth, while the other half was used as a noninduced control (*n* = 3). Differentially expressed genes were defined as those where *P* values were <0.05 (horizonal dashed line in b) and the |fold change| was >2 (outside of vertical gray bar in panel b). (b) Sporulation genes, as defined in SubtiWiki (98), were upregulated more than other genes after induction of a phage-derived sigma factor (ELDg169) and host-derived sigma factors that regulate sporulation (*sigF* and *sigG*). The numbers of upregulated (“up”), downregulated (“down”), and unchanged genes are noted on each panel. Asterisks correspond to significance levels (adjusted for multiple testing) for enrichment of sporulation genes among all genes of each strain using a hypergeometric test (***, *P < *0.001). (c) Correlation of differential gene expression for cells induced to express phage-derived sigma factors (noted above each plot) and cells induced to express bacterium-derived sigma factors (*sigF*). Spearman’s correlation coefficient (*ρ*) is displayed. ELD, phage Eldridge.

We hypothesized that phage-encoded homologs can induce transcription of sporulation genes in a bacterial host. We tested this by cloning four sporulation-like sigma factors (Table 1) from three *Bacillus* phages under an IPTG (isopropyl-β-d-thiogalactopyranoside)-inducible promoter into an ectopic site in the chromosome of a spore-forming strain of B. subtilis. For comparison, we independently cloned the host-encoded sigma factors *sigF* and *sigG* into the same strain of B. subtilis in a similar manner. We then induced the expression of each of the cloned sigma factors during exponential growth (Fig. 3a), a time when native sporulation-specific sigma factors (i.e., *sigF*, *sigG*, *sigE*, and *sigK*) and other sporulation genes are not typically expressed (see Fig. S2). Using RNA-seq (transcriptome sequencing), we compared the transcriptional activity of induced and noninduced cells in paired cultures (68, 69). We found that induction of host-derived *sigF* and *sigG* in log-phase cells resulted in differential expression of more than 1,000 genes with a significant upregulation of more than 300 sporulation-related genes (Fig. 3b; *P < *0.0001; see also Table S1). We documented a similar transcriptional response following induction of one of the phage-derived sigma factors. Specifically, induction of ELDg169 from phage Eldridge resulted in the differential expression of nearly 2,000 host genes along with the upregulation of more than 400 sporulation genes (Fig. 3b; *P < *0.0001; see also Table S1). When considering differential expression across all genes, we found a strong positive correlation (ρ = 0.76) between the transcriptional profiles of cultures where ELDg169 was induced and those where host-derived sigma factors (*sigF* and *sigG*) were induced (Fig. 3b; see also Fig. S3). Induction of a second sporulation-like sigma factor from phage Eldridge (ELDg168) also resulted in differential host expression. However, the transcriptional profile was distinct from that of host-derived sigma factors (*P < *0.0001; see Table S1) and did not include the enrichment of sporulation genes (Fig. 3b). Meanwhile, induction of the two sporulation-like sigma factors from other phages (g120 from SP10 and g157 from Goe3) had only modest effects on gene expression, with less than 50 genes differentially expressed by each induced homolog, and no enrichment of sporulation genes (Fig. 3c; see also Table S1).

**TABLE 1:** Source and characteristics of sigma factors genes cloned in this study

Source	Gene	TIGR match	%ID to:	
			*sigF*	*sigG*
Phage Eldridge	g168	*sigF*	31.67	28.38
	g169	*sigF*	61.64	45.21
Phage SP10	g120	*sigF*	23.83	23.36
Phage Goe3	g157	*sigG*	27.15	25.68
B. subtilis	*sigF*	*sigF*	100	48.42
	*sigG*	*sigG*	48.42	100

10.1128/msphere.00297-22.2 FIG S2 Sporulation gene expression is low in log-phase Bacillus subtilis cultures. The distribution of RNA-seq reads mapped to all B. subtilis Δ6 genes (after normalizing for gene length) is shown by the black curve, while the color-filled curves separate the data into sporulation genes and nonsporulation genes. Each panel shows data for a single culture of the control strain containing the IPTG-inducible promoter with no coding sequence. Each of the three cultures (r1 to r3) was grown to mid-log phase in LB medium before splitting the culture and adding IPTG to one half and water to the other half (“NT” = negative treatment). After two additional hours under growth conditions, cultures were harvested for RNA extraction and sequencing. The small peak on the left represents genes with zero reads, as normalization was done on the number of reads + 1 to allow depiction of zero values on a logarithmic scale. Download FIG S2, PDF file, 0.3 MB. Copyright © 2022 Schwartz et al. 2022 Schwartz et al. https://creativecommons.org/licenses/by/4.0/ This content is distributed under the terms of the Creative Commons Attribution 4.0 International license.

10.1128/msphere.00297-22.3 FIG S3 Supplement to main RNA-seq figure (Fig. 3c). Correlation of differential gene expression for cells induced to express phage-encoded sigma factors (noted above each plot) and cells induced to express bacterium-encoded sigma factor (*sigG*). Spearman’s correlation coefficient (*ρ*) is displayed. ELD, phage Eldridge. Download FIG S3, PDF file, 0.2 MB. Copyright © 2022 Schwartz et al. 2022 Schwartz et al. https://creativecommons.org/licenses/by/4.0/ This content is distributed under the terms of the Creative Commons Attribution 4.0 International license.

10.1128/msphere.00297-22.7 TABLE S1 Enrichment of sporulation genes among differentially expressed genes. Download Table S1, PDF file, 0.2 MB. Copyright © 2022 Schwartz et al. 2022 Schwartz et al. https://creativecommons.org/licenses/by/4.0/ This content is distributed under the terms of the Creative Commons Attribution 4.0 International license.

### Phage-encoded sigma factors inhibit sporulation.

**FIG 4:**
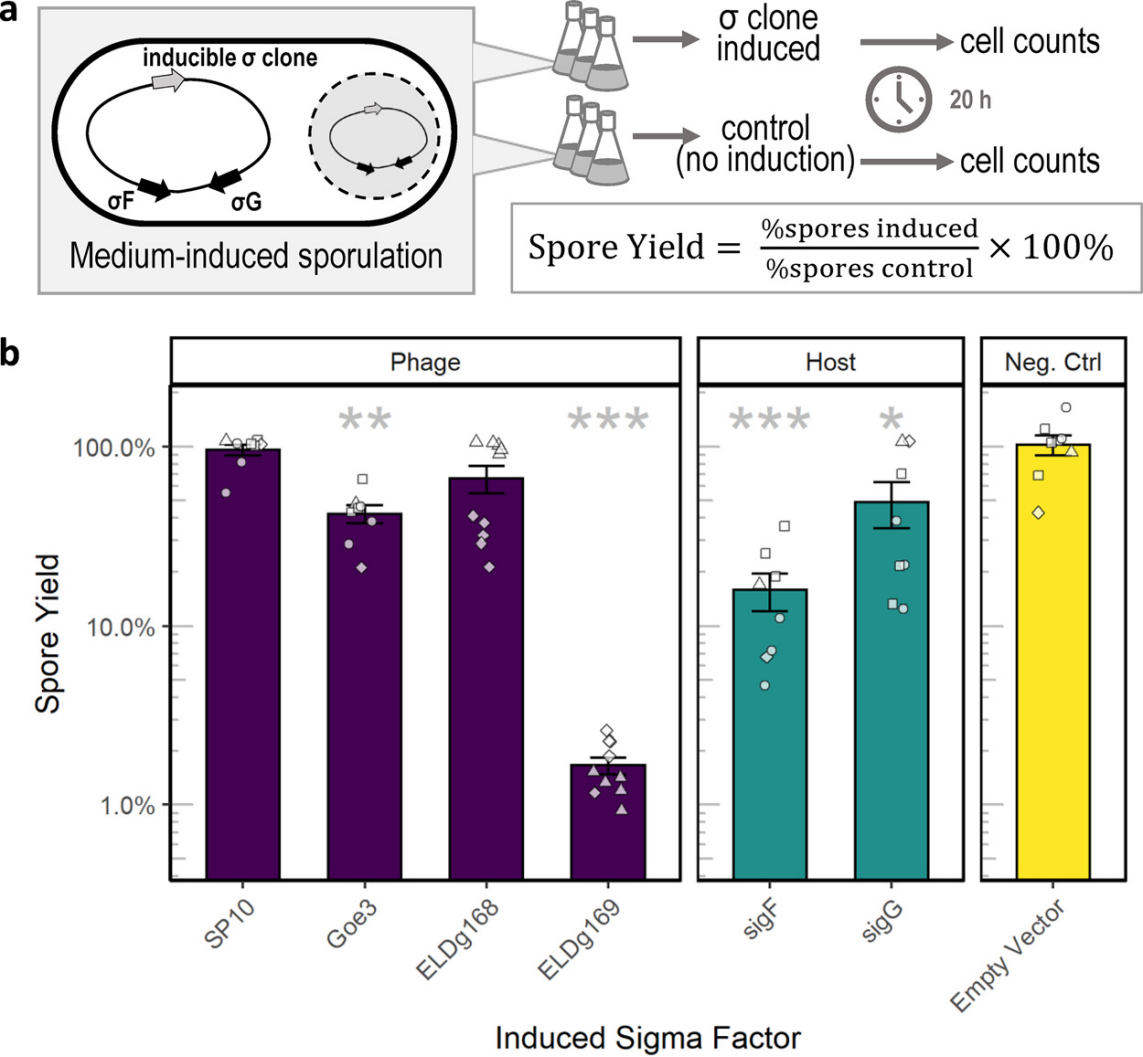
Phage-derived sigma factors reduced sporulation. (a) Illustration of the experimental design. Phage- and host-derived sigma factors were cloned in B. subtilis under an IPTG-inducible promoter (gray arrow) in a strain (Δ6) that contains a fully functional sporulation gene network, including its native sporulation-specific sigma factors (black arrows depict representative examples). We compared sporulation in these strains to an empty-vector negative-control strain that also had an IPTG-inducible promoter. For each data point in panel b, replicate cultures (*n* = 6) of a single colony were grown in sporulation medium for 4.5 h, a time at which sporulation was induced by nutrient exhaustion. We then induced expression of the cloned sigma factor by the addition of IPTG to half of the replicates, while leaving the remainder as controls. Spore and vegetative cells were quantified by flow cytometry. Spore yield was calculated as the ratio of percent spores in induced cultures and their paired controls. In panel b, bars represent the means ± the SEM of independent clones (*n* ≥ 8). Shapes represent different experimental batches. Asterisks correspond to significance levels (adjusted for multiple testing) from Welch’s *t* tests used to evaluate the effect of each sigma factor compared to the empty-vector negative control (*, *P < *0.05; **, *P < *0.01; ***, *P < *0.001). ELD, phage Eldridge.

We tested whether changes in transcription associated with the induction of sporulation-like sigma factors could lead to altered spore yield. We cultivated B. subtilis strains under conditions that promote sporulation (see Materials and Methods) and induced the expression of the cloned sigma factors (Table 1) at the onset of sporulation (see Fig. S4a). After 24 h, we quantified changes in spore yield for cultures that were induced to express a cloned sigma factor and for noninduced cultures (Fig. 4a). Compared to a to an empty vector strain (with no cloned sigma factor), induction of *sigF* led to an 85% reduction in spore yield (*t*_8.2_ = 6.43, *P < *0.001) while the induction of *sigG* led to 50% reduction in spore yield (*t*_13.9_ = 6.43, *P = *0.015). The expression of phage-derived sigma factors also reduced sporulation, but to different extents (Fig. 4; see also Table S2a). Induction of ELDg169 reduced the spore yield by 99% compared to the empty vector control (*t*_7_ = 7.8, *P < *0.0001), while the second sporulation-like sigma factor in Eldridge phage (ELDg168) had a smaller (33%) and marginal effect on spore yield (*t*_15.1_ = 2.1, *P = *0.064). The sigma factor from phage Goe3 reduced spore yield by > 50% (*t*_8.9_ = 4.39, *P = *0.002), while expression of the sigma factor from phage SP10 had no effect on spore yield (*t*_10.4_ = 0.46, *P = *0.65). Because spore yield was calculated as a percentage of the total population, we compared cell counts between induced and noninduced controls (see Fig. S4b). From this, we concluded that the observed reductions in spore yield were not due to a reduction in vegetative cells (see Table S2b).

10.1128/msphere.00297-22.4 FIG S4 B. subtilis densities in sporulation assays. Sporulation was measured in strains with phage-derived or bacterium-derived (host) sigma factors under an IPTG-inducible promoter. The empty-vector negative control had the IPTG-inducible promoter without any sigma factor. (a) Growth of B. subtilis during sporulation assays. Each panel shows the growth (means ± the SEM, *n* = 3) measured as the optical density (OD_600_) over time of cultures with cloned sigma factor (row label on the right). Column numbers represent independent colonies from which experimental cultures were inoculated. Cultures were grown for 4.5 h (gray vertical line) in sporulation medium before IPTG was added to half of them. Samples for quantifying spores and vegetative cells by flow cytometry were taken at 24 h. (b) Effect of sigma factor induction on endpoint cell density. Cells were quantified and classified by flow cytometry after 24 h (spores + vegetative = total cells). Points connected by thick lines represent means ± the SEM of independent colonies (*n* ≥ 8). Thin lines connect values of each induced clone and its noninduced paired control. Download FIG S4, PDF file, 0.5 MB. Copyright © 2022 Schwartz et al. 2022 Schwartz et al. https://creativecommons.org/licenses/by/4.0/ This content is distributed under the terms of the Creative Commons Attribution 4.0 International license.

10.1128/msphere.00297-22.8 TABLE S2 Statistical results for sporulation of cells expressing cloned sigma factors. (a) Spore yield statistics. We used flow cytometry to quantify spores and vegetative cells. With these data, we calculated the fraction of spores in cultures of B. subtilis strains with sigma factors cloned under an IPTG-inducible promoter (indicated in “group 1” column), or with empty vector controls (“group 2”). The mean value of each group is the ratio of spore fraction in induced versus noninduced cultures (*n* > 8). We used a two-tailed Welch two Sample *t*-test, (difference in means ≠ 0) to compare cloned gene effect on sporulation between each of the cloned genes and the empty vector controls. (b) Statistics associated with total cell densities following sigma factor induction. We used flow cytometry to quantify spores and vegetative cells in B. subtilis strains with cloned sigma factor genes. We used a two-tailed one sample *t* test (mean ≠ 0) to compare the difference in cell densities between induced and noninduced paired samples. Download Table S2, PDF file, 0.3 MB. Copyright © 2022 Schwartz et al. 2022 Schwartz et al. https://creativecommons.org/licenses/by/4.0/ This content is distributed under the terms of the Creative Commons Attribution 4.0 International license.

## DISCUSSION

Our study was motivated by the detection of sporulation-like sigma factors in phage genomes. For bacteria that engage in endosporulation, alternate sigma-factors are key regulators of gene expression that are required for transitioning into a dormant state. Some phages also have sigma factors. While most commonly used to regulate gene expression during reproduction, it is possible that alternate sigma factors could be used by phages for other purposes. We speculated that sporulation-specific sigma factors may be coopted by phages to manipulate host dormancy. In support of this hypothesis, our bioinformatic analyses revealed that sporulation-like sigma factors were restricted to phages that infect spore-forming hosts, where they could potentially interfere with the regulation of the host’s sporulation gene network. Furthermore, our phylogenetic evidence demonstrates that phage-encoded sigma factors are distinct from genes known to be essential for regulating lytic reproduction. In support of this pattern, we experimentally demonstrate that, in at least one case, a sporulation-like sigma factor is nonessential for phage reproduction. Finally, when expressed in Bacillus subtilis, we show that phage-encoded sigma factors can lead to the transcriptional activation of the sporulation network and the reduction of spore yield. Together, our findings highlight novel ways in which dormancy may influence antagonistic coevolution between spore-forming bacteria and their phages.

Sigma factors are not found in all phage genomes. In fact, less than 15% of >4,000 phage genomes in the VOG database contained a sigma-factor homolog. Some of these sigma factors are involved in the expression of genes required for phage development (36, 52, 70). Because this is a common function, it is perhaps not surprising that sigma-factor homologs were found in phages that infect a diverse and broad range of bacteria (Fig. 1a). In contrast, the host range of phages containing sporulation-like sigma factors was much more constrained. In all cases, sporulation-like sigma factors were found in phages that infected hosts belonging to clades of endospore-forming bacteria. Such a distribution suggests that phages most likely acquired these alternative sigma factors from their spore-forming hosts. The occurrence of sporulation-like sigma factors in dozens of phages from diverse vial families raises the question of whether these homologs might confer a benefit to phages that carry them. It is possible that alternative sigma factors could be acquired by phage and used in their reproduction programs. It is also possible that alternate sigma factors retain their ancestral function. In this way, phage-encoded sporulation-like sigma factors could affect the regulation of host genes required for the development of endospores. If so, dormancy related functions could be added to the growing list of host-acquired auxiliary genes that can affect phage fitness (25, 63).

Sigma factors are essential when used by phages to control core viral functions. For example, the late transcription sigma factor of E. coli phage T4 (gp55) is essential for productive infection (70). Likewise, sigma factors gp28 and gp34, which regulate middle and late infection genes in phage SPO1, are indispensable for infection of B. subtilis (36). However, multiple lines of evidence suggest that sporulation-like sigma factors are nonessential for fundamental aspects of phage biology. A sporulation-like sigma factor in SPO1 (gp2.21) has been shown to be nonessential for lytic infection under standard laboratory conditions (36). While it has been hypothesized that gp2.21 directs transcription of phage genes from *sigK*-like promoters found in its genome (36), this is yet to be confirmed. Like SPO1, phage SP10 encodes three sigma factors: g183 and g200 are homologs of the SPO1 middle and late sigma factors (65), respectively, while the third is a sporulation-like sigma factor (g120). In our study, we deleted g120 from phage SP10 with no observable effect on virulence (Fig. 2). Even though g120 is expressed during SP10 infection of its host (65), evidence suggests that it is nonessential for lytic infection. We attempted to delete sporulation-like sigma factors in two other phages (Goe3 and Eldridge), but encountered technical obstacles associated with cloning and host susceptibility. The development of genetic systems for phage that infect other bacteria besides B. subtilis, will provide much needed insight into the sporulation-like sigma factors of phage Eldridge, and others. In addition to making generalizations about essentiality, these genetic systems will provide tools for characterizing the functionality of alternate sigma factors, including the ability of phages to manipulate sporulation in ways that would affect their fitness.

Given their prevalence among diverse phages along with evidence of nonessentiality, it is possible that sporulation-like sigma factors are auxiliary genes that can alter sporulation in bacteria. Previous experiments have shown that phage-encoded sporulation-like sigma factors retain a function that is relevant to host sporulation. For example, in lysogenic B. anthracis, expression of a sporulation-like sigma factor encoded by the Wβ prophage is elevated during sporulation (42). In addition, *in vitro* reconstitution of RNA polymerase with the sporulation-like sigma factor of B. anthracis phage Fah, a close relative of Wβ, revealed patterns of transcriptional activity and inhibition that are similar to that of a *sigF*, a bacterium-encoded sigma factor that is expressed during the early stages of spore development (58). Finally, sporulation-like sigma factors cloned from two diverse B. anthracis phages, Bcp1 (*Herelleviridae*) and Wip4 (*Siphoviridae*), were associated with phage-dependent inhibition of host sporulation (41).

Our results reveal a sporulation-related role for the ELDg169 sigma factor of phage Eldridge. When we induced ELDg169 in B. subtilis, the functional responses were nearly indistinguishable from when the native *sigF* or *sigG* was induced in the same manner. Specifically, there was a strong correlation across the bacterial genome in both the direction and magnitude differential gene expression, including the upregulation of hundreds of sporulation-related genes (Fig. 3). Furthermore, we documented a significant reduction in spore yield when ELDg169, *sigF*, and *sigG* were independently expressed in B. subtilis (Fig. 4). These results indicate that ELDg169 has the potential to act as an analog of host-encoded sporulation-specific sigma factors. When similar experiments were run with phage-derived homologs (ELDg168 and Goe3 g157) that are more divergent from *sigF* and *sigG*, we observed a less pronounced reduction in spore yield, and almost no effect on the expression of sporulation genes (Fig. 4). Similarly, the induction of g120 from phage SP10 had a minor effect on host transcription and no effect on spore yield, consistent with it being most divergent from native sigma factors (*sigF* and *sigG*). Taken together, our results suggest that the functionality of phage-encoded sigma factors can be predicted by sequence similarity with host-encoded sigma factors (see Fig. S5).

10.1128/msphere.00297-22.5 FIG S5 Sporulation response corresponds with the identity of sigma factors induced in B. subtilis. (a) Multiple sequence alignment of sigma factors cloned in this study. Protein sequences from phages and from B. subtilis (Bsub) were aligned to the protein profile of the RNA polymerase sigma-70 factor, sigma-B/F/G subfamily (TIGR02980) using hmmalign (HMMER v3.3). The profile reference positions are marked with ‘x’ at the bottom of the alignment. Functional sequence regions of the host sigma factors are depicted above the alignment by color bars: region 2 in blue, region 3 in red, and region 4 in green, with the helix-turn-helix motif marked by a black box. Functional region annotation from SWISS-MODEL data for B. subtilis genes *sigF* (P07860) and *sigG* (P19940). (b) Protein identities from the alignment. The identities were calculated using esl-alipid (part of the Easel library of HMMER v3.3) after trimming the N and C termini beyond the profile reference positions (using hmmalign –trim). (c and d) The amino acid identity between cloned sigma factors and B. subtilis *sigF* is negatively correlated with the mean spore yield (c) (see Fig. 4) when induced during sporulation and positively correlated with the number of upregulated sporulation genes (d) when induced in exponential phase (see Fig. 3). Spearman’s rho (ρ) and the associated *P* value (*P*), and linear model (dashed line) were calculated in R. The error bars in panel c represent the SEM. Download FIG S5, PDF file, 0.7 MB. Copyright © 2022 Schwartz et al. 2022 Schwartz et al. https://creativecommons.org/licenses/by/4.0/ This content is distributed under the terms of the Creative Commons Attribution 4.0 International license.

Our study demonstrates that there is functional conservation for at least some phage-encoded sigma factors. With our experiments, we were able to compare the effects of inducing phage and host genes from the same promoter under identical genetic and cellular conditions. Yet, an argument can be made that the conditions under which the experiments were conducted are somewhat disconnected from their ecological context. Our approach involved expression of genes at times and in cellular compartments that differ from natural conditions. Furthermore, induction may have indirectly altered expression profiles by competitively excluding other sigma factors from binding to RNA polymerase (71, 72). This likely explains why spore yield decreased when we induced the expression of host sigma factors whose known function is the promotion of sporulation. Nevertheless, the effect of phage-derived sigma factors was reproducible and led to a significant enrichment in upregulated sporulation genes, which would not be predicted from a generalized disruption of cell-wide transcription.

The function of phage-encoded sporulation-like sigma factors in the infected cell remains to be determined. We initially suspected that sporulation-like sigma factors may be used to manipulate the progression of sporulation so as to increase their entrapment and increase phage survivorship (46). Alternatively, the expression of phage-encoded sigma factors could disrupt sporulation, creating more opportunities for virus replication thus increasing the reproductive fitness of phages. The results from our study are more consistent with the latter scenario. Despite the limitations discussed above, our study demonstrates that the expression of these alternate sigma factors phages could inhibit sporulation. Furthermore, the two Eldridge-derived genes (ELDg168 and ELDg169), which are genomic neighbors, both reduced sporulation, even though they had different transcriptional profiles (Fig. 4). If these adjacent genes work toward a common function, it may be the inhibition of spore development. This interpretation is consistent with the observation that complete inhibition of sporulation in B. anthracis is apparently mediated by a pair of sporulation-like sigma factors found in tandem in the genome of phage Bcp1 (41). These findings do not rule out that sporulation-like sigma factors may play other roles during infection. In particular, it will be valuable to compare host-phage dynamics where sporulation-like sigma factors have been genetically deleted. The findings from such experiments would provide a direct test for determining whether or not auxiliary dormancy genes have consequences for phage fitness. While we created this type of a deletion mutant for phage SP10, our functional analyses do not support a sporulation-related role for this particular phage (Fig. 3 and 4). Likewise, comparative transcriptomic analysis during infection with phages with or without sigma factors under various environmental conditions may help identify the regulatory targets of phage-encoded sigma factors. Looking beyond sporulation, studies are needed to better understand how bacterium-like sigma factors may be used by phages to manipulate other survival strategies that are common in nongrowing bacteria (73).

As obligate parasites, phages are unavoidably dependent on the metabolism of their bacterial hosts. Bacteria are capable of responding to fluctuations in their environment by replacing the sigma subunit of RNA polymerase, which leads to changes in gene expression (51, 52). Some phages use a similar strategy to coordinate expression of their own genes during different stages of infection (56). Our analysis points to the existence of a second class of sigma factors that phages may use to manipulate host metabolism. Whether phages promote or inhibit sporulation, such manipulation of host dormancy has the potential to modify the environmental conditions under which bacteria transition between active and inactive states. This has implications for development of novel therapeutic treatments that combine phage therapy with antimicrobials, which tend to target metabolically active bacteria (74). More generally, the discovery of virus-encoded auxiliary metabolic genes has revealed that exploitation of a host by viruses is not limited to cellular building blocks or the appropriation of the protein translation machinery. Viruses have evolved to use, maintain, and rearrange a variety of biochemical pathways in the cells that they take over (25, 28, 63). Our findings highlight an additional aspect of phage-host coevolution, that is, the co-option of gene networks that allow microorganisms to contend with harsh and unpredictable environments. Manipulation of the host’s response to such conditions through the acquisition of host regulatory genes could represent a strategy that buffers viruses from the dynamic cellular environment on which their survival and reproduction is dependent.

## MATERIALS AND METHODS

### Phage sigma factor distribution and classification.

We retrieved sigma factors from the database of viral orthologous groups (VOG release vog209, vogdb.org) based on text searches of the VOG descriptors (see Table S3). We clustered the phage genomes used to construct the VOG database into “virus operational taxonomic units” using CD-HIT-est (75) set to recommended thresholds (76). VOG phages were matched to host and viral taxonomy using the virus-host database (77). We classified phage-encoded sigma factors using hmmscan with default parameter settings using HMMER v3.3 (78), and queried each protein against hidden Markov model (HMM) profiles of bacterium-encoded sigma factor families that were retrieved from TIGRFAM (see Table S3). We used the best hmmscan match (smallest sequence E value) to classify proteins, unless it was a general TIGRFAM (“sigma70-ECF” or “SigBFG”), in which case the next best match was chosen, if available. To ensure the bacterial nature of the TIGRFAM profiles we traced the origins of the TIGRFAM seed proteins to check if any had from phages or prophages. To identify prophage regions, we used PHASTER (79). Of the 372 (of 556) seed proteins that we could trace to their gnomic origin, only 7 were from phages. However, the only TIGRFAM that contained phage sigma factor proteins in its seed was the most general super family (“sigma70-ECF,” TIGR02937). Our analysis was able to identify the origins of all but one of the seeds used to construct the sporulation-specific sigma TIGRFAMs, and none were from phages.

10.1128/msphere.00297-22.9 TABLE S3 Sigma factor protein groups of homologs. (a) Viral orthologous groups (VOG) of sigma factors. (b) TIGRFAM protein families of bacterial sigma factors. Download Table S3, PDF file, 0.2 MB. Copyright © 2022 Schwartz et al. 2022 Schwartz et al. https://creativecommons.org/licenses/by/4.0/ This content is distributed under the terms of the Creative Commons Attribution 4.0 International license.

### Phylogenetic analysis.

For phylogenetic analysis of sigma factors, we clustered VOG proteins from phage genomes at 95% identity using CD-HIT-est (75). Cluster representatives were aligned along with sigma factor protein sequences belonging to 24 bacteria from diverse taxa (80). For the alignment, we used MAFFT (v.7.49) (81) with the E-INS-I strategy and trimmed the alignment with trimAL (v1.4.rev22) (82) using the gappyout method. From the 193 amino acids in the trimmed alignment, we then inferred 500 maximum-likelihood phylogenetic trees using RAxML-NG (v0.9.0-pthreads) (83) with the LG+G4 substitution model selected using modeltest-NG (v0.1.6) (84) with default settings. We present the best scoring maximum likelihood tree with Transfer Bootstrap Expectation supports (85) from 1,000 bootstrapped trees. We plotted the tree using the ggtreeExtra R package (86).

### Strains and media.

Strains used in our study are listed in Table S4. For routine culturing of bacteria, we used Lysogeny Broth (LB) medium with low salt (5 g/L NaCl). We amended this recipe with agar (15 g/L) for plating and with CaCl_2_ (10 mM) to facilitate virus adsorption. We used Difco sporulation media (DSM) for sporulation assays (87). For plaque assays, we used double-layer plating with 0.3% agar overlays (88). To amplify phages, we collected lysates from plate infections after flooding petri dishes with phage buffer (10 mM Tris, 10 mM MgSO_4_, 4 g/L NaCl, 1 mM CaCl_2_ [pH 7.5]). We then cleared the phage-containing buffer from bacteria by centrifugation (7,200 × *g*, 10 min) and filtration (0.2 μm).

10.1128/msphere.00297-22.10 TABLE S4 Strains, plasmids, and primers used in this study. (a) Bacterium and phage strains. (b) Plasmids. (c) Primers. Download Table S4, DOCX file, 0.02 MB. Copyright © 2022 Schwartz et al. 2022 Schwartz et al. https://creativecommons.org/licenses/by/4.0/ This content is distributed under the terms of the Creative Commons Attribution 4.0 International license.

### Deletion of phage-encoded sigma factor.

We used the CRISPR-Cas9 system and the CutSPR assay design-tool (64) to test whether sigma factors are essential for phage replication. Briefly, we cloned a single-guide RNA and a deletion cassette into plasmid pJOE8999 (89) (Table S1) and transformed the resulting plasmid into B. subtilis TS01 (see Table S4), which was made competent with d-mannitol induction. We next infected the transformed culture with phage SP10 (see Table S4) and conducted a plaque assay with medium containing the Cas9-inducer d-mannose. Using the primers SP10_validF+R (see Table S4), we screened multiple plaques for the deletion. To isolate the mutant phages, we picked and replated PCR-positive plaques onto host B. subtilis Δ6 (90) (see Table S4). We screened these secondary plaques as described above and confirmed the deletion by Sanger sequencing of the locus. We then quantified the virulence of the mutant and wild-type phages (91). After dispensing B. subtilis Δ6 host cultures (optical density at 600 nm [OD_600_] = 1) into microtiter wells, we infected cells with serially diluted lysates of SP10 or SP10 Δg120 that were adjusted to an equal titer. We monitored bacterial density during growth for 16 h by determining the OD_600_ with a Synergy H1 plate reader (BioTek). From this, we calculated the virulence index (91) based on change in bacterial growth and lysis as a function of the phage/bacterium ratio (i.e., multiplicity of infection; Fig. 2a).

### Inducible expression of sigma factors.

We tested the effect of phage-derived sigma factors on bacterial expression by cloning coding sequences under an inducible promoter into an ectopic site (*amyE*) of the B. subtilis genome. As a control, we also cloned host-derived sporulation genes (s*igF* and *sigG*) in the same manner, and a gene-less promoter as a negative control.

### (i) Strain construction.

We amplified coding sequences by PCR from phage lysates or from extracted bacterial genomic DNA as templates using primers adapted with restriction sites, and a ribosome binding site on the forward primer (see Table S4). We then cloned the PCR products into plasmid pDR110 (see Table S4) by restriction enzyme digestion (see Table S4), gel purification, and ligation (T4 ligase). We selected for plasmids that were transformed into E. coli (One Shot TOP10; Fisher) with ampicillin (100 μg/mL) and verified the insertion by PCR and Sanger sequencing using primers oDAS9+10 (see Table S4). We transformed purified plasmids (Qiagen mini prep) into B. subtilis TS01, as described above, using spectinomycin selection (100 μg/mL). We verified the insertion into the *amyE* locus by PCR and Sanger sequencing and by the loss of erythromycin resistance carried in the *amyE* locus by strain TS01.

### (ii) Transcriptional response to phage-encoded sigma factor.

We diluted overnight B. subtilis cultures (OD_600_ = 0.1) in fresh LB medium and grew them (37°C, 200 rpm) to midexponential phase (OD_600_ = 0.5). We then split the cultures and added 1 mM IPTG (final concentration) to one half to induce expression of the cloned gene. We added an equal volume of water to the other half of the split culture as a noninduced control. After induction, we incubated the cultures for 2 h before harvesting cells. Upon sampling, we immediately treated bacteria with the RNAprotect Bacteria Reagent (Qiagen) and stored pellets at −80°C for <1 week before RNA extraction using RNeasy Protect Bacteria minikit (Qiagen) according to the manufacturer’s instructions (protocol 5), including an on-column RNase-free DNase digestion. Library construction, sequencing, and analysis of differential gene expression were all carried out at the Indiana University Center for Genomics and Bioinformatics. Libraries were constructed using the Illumina TruSeq Stranded mRNA HT kit following depletion of rRNA using Illumina Ribo-Zero Plus kit. Libraries were then sequenced on an Illumina NextSeq 500 platform as paired-end reads (2 × 38 bp). We trimmed adapters and filtered reads using Trimmomatic 0.38 (92) with the cutoff threshold for average base quality score set at 20 over a window of three bases. Reads shorter than 20 bases posttrimming were excluded. We mapped the cleaned reads to the reference genome (deposited with sequencing data to the Gene Expression Omnibus; see below) using bowtie2 version 2.3.2 (93) and counted reads that were mapped concordantly and uniquely to the annotated genes using featureCounts tool v2.0.0 of the subread package (94). Read alignments to antisense strand, or to multiple regions on the genome or those overlapping with multiple genes were ignored (parameters: -s 2 -p -B -C). We performed differential expression analysis using DESeq2 v1.24.0 (95) from normalized read counts by comparing samples induced with IPTG to noninduced paired control samples, with multiple-testing correction by the Benjamini, Hochberg, and Yekutieli method. We tested for the effects of gene enrichment and overlap of differentially expressed genes using the hypergeometric distribution in R (96).

### (iii) Sporulation of cells expressing cloned sigma factors.

To test for the effects of induced sigma factors on host sporulation, we diluted overnight B. subtilis cultures in fresh DSM (OD_600_ = 0.05) and dispensed each culture into multiple wells of a 96-well plate that was then incubated in a BioTek Synergy H1 plate reader (37°C, fast and continuous shake setting). Under these conditions, we determined that cells enter stationary phase after approximately 4.5 h, marking the onset of sporulation. At this time, we induced expression of the cloned gene by adding IPTG (final concentration 1 mM) to half the cultures in the plate. We added water to the rest of the wells, which served as noninduced controls. At 24 h, we quantified the number of spores and vegetative cells in each well using a flow cytometry assay that distinguished spores from vegetative cells (nonspores) based on differential uptake of the nucleic acid stain SYBR green (97). We diluted each sample in Tris-EDTA buffer (pH 8) and then fixed the cells in 0.5% glutaraldehyde for 15 min at 4°C. We stained the fixed samples with SYBR green (20,000× dilution of commercial stock, Lonza) for 10 min at room temperature in the dark. We then enumerated cells using a volumetric NovoCyte 2000R flow cytometer (Acea; excitation, 488 nm; emission, 530/30 nm) and an automatic gating pipeline (see Fig. S6).

10.1128/msphere.00297-22.6 FIG S6 Gating strategy used to quantify B. subtilis spores and vegetative cells. (a to d) Analysis was done on cells treated with DNA stain SYBR green that can penetrate vegetative cells, but not spores. Gating was done using an automated pipeline after the scattering, and fluorescence signals were normalized by transformation with the hyperbolic arcsine (“asinh”) function. (a) Singlets were separated from doublets using the gate_singlet function of the flowStats R package applied to the transformed height (“h”) and area (“a”) of the forward scatter (FSC) signal intensity. (b) Events attributed to noise (not cells) were identified as the lower tail of the distribution of transformed FSC area values in each sample using the rangeGate function of the flowStats package in R. (c) Noise gate applied to singlets. (d) To cluster events into vegetative and spore populations, we constructed a two population Gaussian mixture model using noninduced control samples for each strain on each experimental run, using the no-IPTG control samples. The models were built using the Mclust function of the mclust R package based on the transformed data of the FSC and the SYBR green fluorescence intensity areas. The mean SYBR green intensity of the model populations (blue dots) was used to assign populations as vegetative (high SYBR) and spores (low SYBR). The model was then applied to classify events in all samples of the strain as spore and vegetative cells. The example sample is from experiment 4 and shows a single culture (well F7) of the empty vector control strain, with no IPTG treatment. Download FIG S6, PDF file, 0.5 MB. Copyright © 2022 Schwartz et al. 2022 Schwartz et al. https://creativecommons.org/licenses/by/4.0/ This content is distributed under the terms of the Creative Commons Attribution 4.0 International license.

### Code and data availability.

All code and data used in the analyses in this study are available at github.com/LennonLab/sigma-spore-phage (https://doi.org/10.5281/zenodo.6818421) and github.com/LennonLab/sigma-spore-phage-flow (https://doi.org/10.5281/zenodo.6819190). RNA sequencing data are available at the Gene Expression Omnibus under accession number GSE187004.
