# Supplementary material for: Phage-Encoded Sigma Factors Alter Bacterial Dormancy
Source: mSphere. 2022 Jul 20;7(4):e00297-22. doi: 10.1128/msphere.00297-22 (PMC9429907; doi:10.1128/msphere.00297-22)
Supplement: FIG S1 [file msphere.00297-22-s0001.pdf]

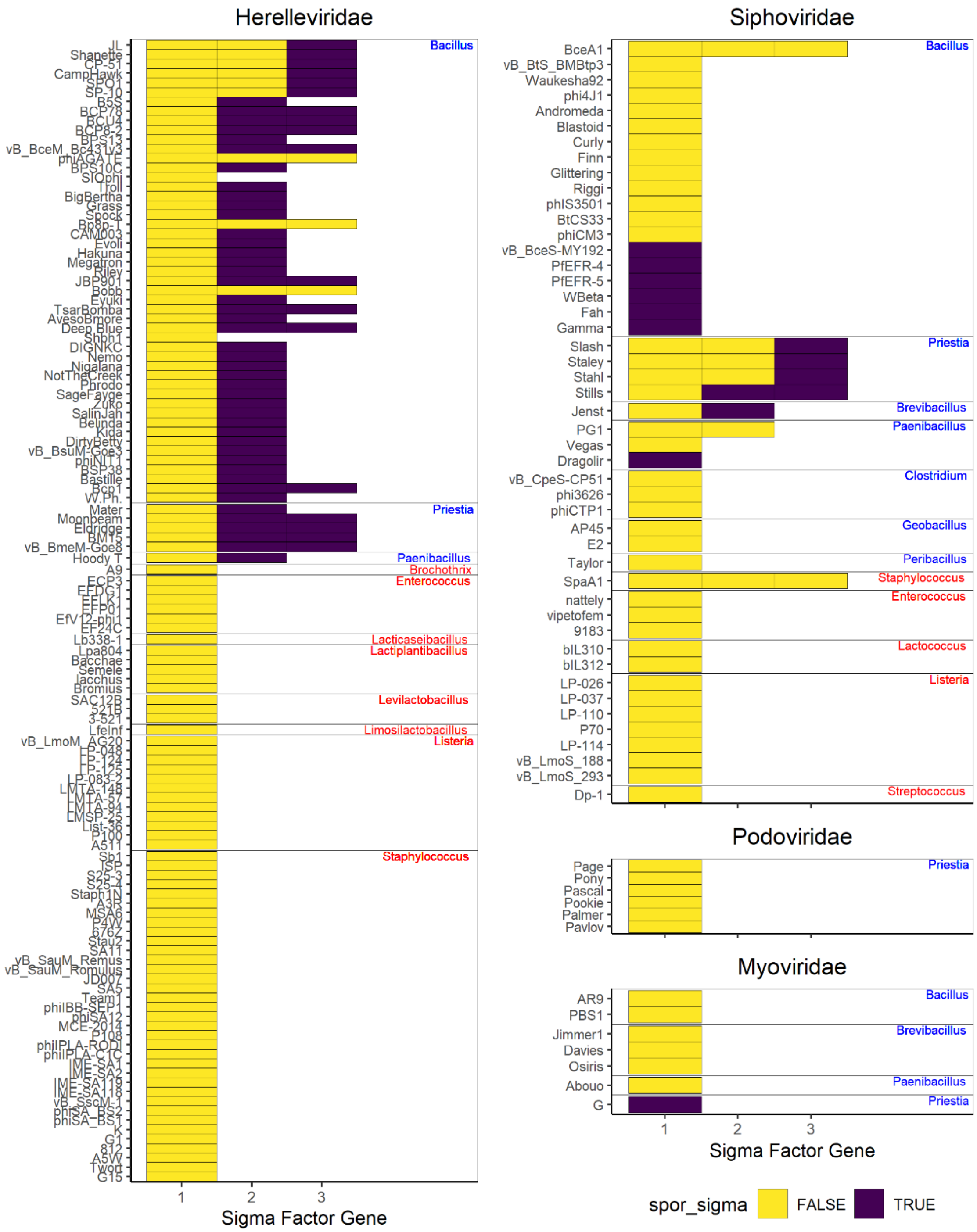

Fig. S1. Distribution of sigma factors in *Bacillota* phages. Each colored rectangle represents a single sigma factor gene encoded by the phage whose name is indicated in the row label. Panels separate phages by viral family (panel titles in black), and the host genera are depicted in the upper right of each sub-panel. Text color of the genus name corresponds to endospore-forming (blue) v. non-endospore-forming (red) genera, as defined by Bergey's manual (Vos et al. 2011. Bergey's manual of systematic bacteriology: Volume 3: The Firmicutes, Springer Science & Business Media.).
