# Supplementary material for: Phage-Encoded Sigma Factors Alter Bacterial Dormancy
Source: mSphere. 2022 Jul 20;7(4):e00297-22. doi: 10.1128/msphere.00297-22 (PMC9429907; doi:10.1128/msphere.00297-22)
Supplement: FIG S2 [file msphere.00297-22-s0002.pdf]

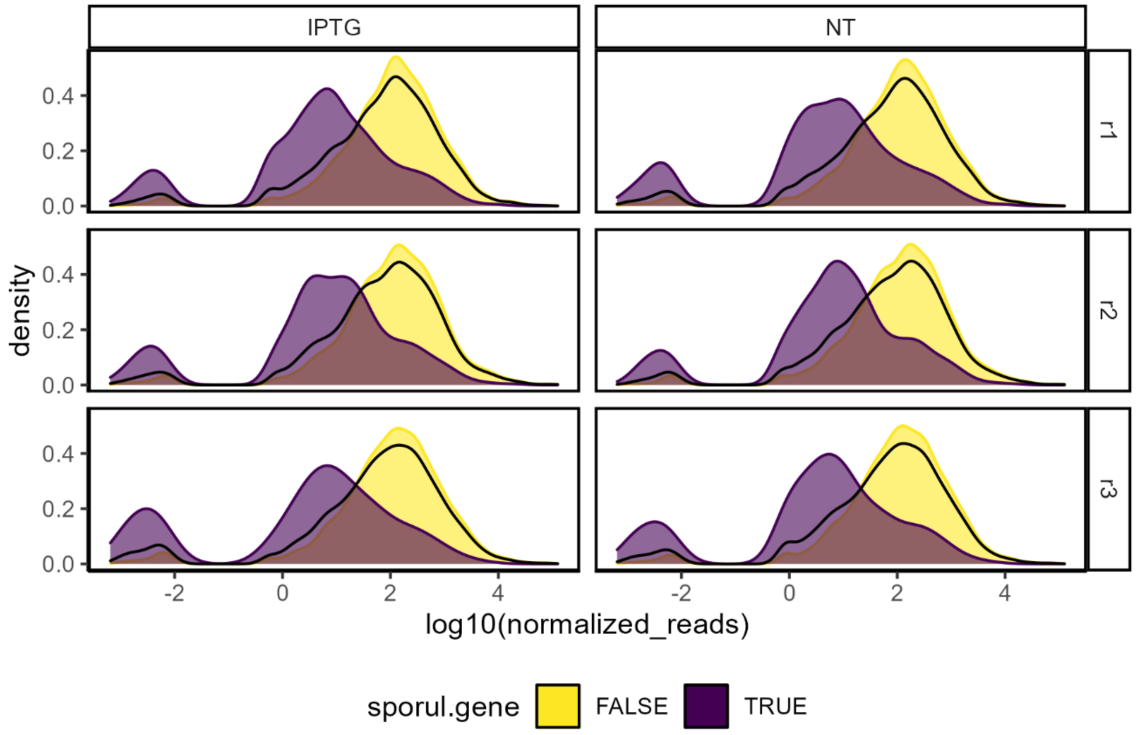

Fig. S2. Sporulation gene expression is low in log-phase *Bacillus subtilis* cultures. The distribution of RNAseq reads mapped to all *B. subtilis* Δ6 genes (after normalizing for gene length) is shown by the black curve, while the color-filled curves separate the data into sporulation genes and non-sporulation genes. Each panel shows data for a single culture of the control strain containing the IPTG-inducible promoter with no coding sequence. Each of the three cultures (r1-3) was grown to mid-log phase in LB before splitting the culture and adding IPTG to one half and water to the other half ("NT" = negative treatment). After two additional hours in growth conditions, cultures were harvested for RNA extraction and sequencing. The small peak on the left represents genes with zero reads, as normalization was done on the number of reads + 1 to allow depiction of zero values on a logarithmic scale.
