# Supplementary material for: Phage-Encoded Sigma Factors Alter Bacterial Dormancy
Source: mSphere. 2022 Jul 20;7(4):e00297-22. doi: 10.1128/msphere.00297-22 (PMC9429907; doi:10.1128/msphere.00297-22)
Supplement: FIG S3 [file msphere.00297-22-s0003.pdf]

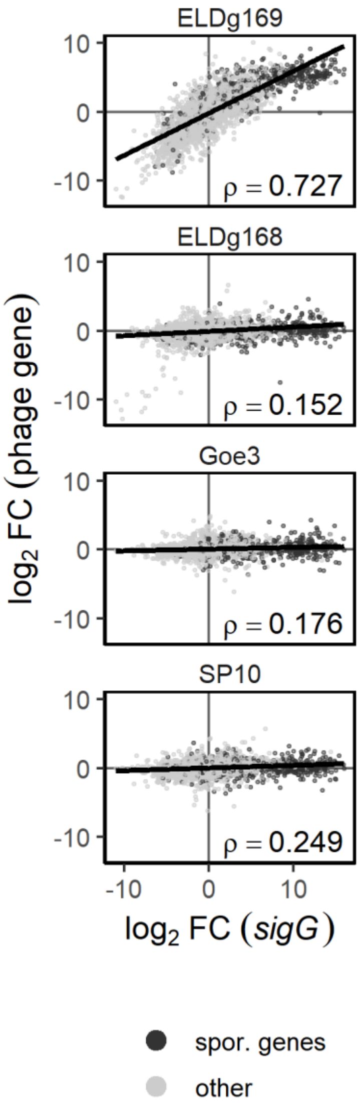

Fig. S3. Supplement to main RNAseq figure (Fig. 3c). Correlation of differential gene expression for cells induced to express phage-encoded sigma factors (noted above each plot) and cells induced to express bacteria-encoded sigma factor (*sigG*). Spearman's correlation coefficient (ρ) is displayed. ELD = phage Eldridge.
