# Supplementary material for: Phage-Encoded Sigma Factors Alter Bacterial Dormancy
Source: mSphere. 2022 Jul 20;7(4):e00297-22. doi: 10.1128/msphere.00297-22 (PMC9429907; doi:10.1128/msphere.00297-22)
Supplement: TABLE S1 [file msphere.00297-22-s0007.pdf]

Table S1. Enrichment of sporulation genes among differentially expressed genes.

Gene induced	Dir. [1]	Differentially Expressed genes		Unchanged genes		Total spor. genes (m) [3]	total other genes (n) [3]	total changed (k) [3]	*P*-value	adjusted *P* [4]
		Spor. [2]	other	Spor. [2]	other					
ELDg168	up	29	193	567	2789	645	3240	222	0.917	1.000
ELDg168	down	49	258	567	2789	645	3240	307	0.587	1.000
ELDg169	up	390	861	192	1291	645	3240	1251	3.27E-60	1.96E-59 ***
ELDg169	down	63	1088	192	1291	645	3240	1151	1.000	1.000
Goe3	up	5	17	640	3221	645	3240	22	0.144	0.433
Goe3	down	0	2	640	3221	645	3240	2	0.305	0.731
sigF	up	338	748	255	1618	645	3240	1086	3.57E-48	1.43E-47 ***
sigF	down	52	874	255	1618	645	3240	926	1.000	1.000
sigG	up	305	491	299	2170	645	3240	796	4.74E-66	5.69E-65 ***
sigG	down	41	579	299	2170	645	3240	620	1.000	1.000
SP10	up	2	28	642	3200	645	3240	30	0.897	1.000
SP10	down	1	12	642	3200	645	3240	13	0.662	1.000

1. Direction of change: upregulated or downregulated.
2. Sporulation related genes, as defined in subtiwiki.uni-goettingen.de.
3. ‘m’, ‘n’ and ‘k’ are parameters of the hypergeometric test (phyper in R)
4. Multiple testing correction by the Benjamini, Hochberg, and Yekutieli method (*** = $P < 0.001$)
