# Supplementary material for: Phage-Encoded Sigma Factors Alter Bacterial Dormancy
Source: mSphere. 2022 Jul 20;7(4):e00297-22. doi: 10.1128/msphere.00297-22 (PMC9429907; doi:10.1128/msphere.00297-22)
Supplement: FIG S4 [file msphere.00297-22-s0004.pdf]

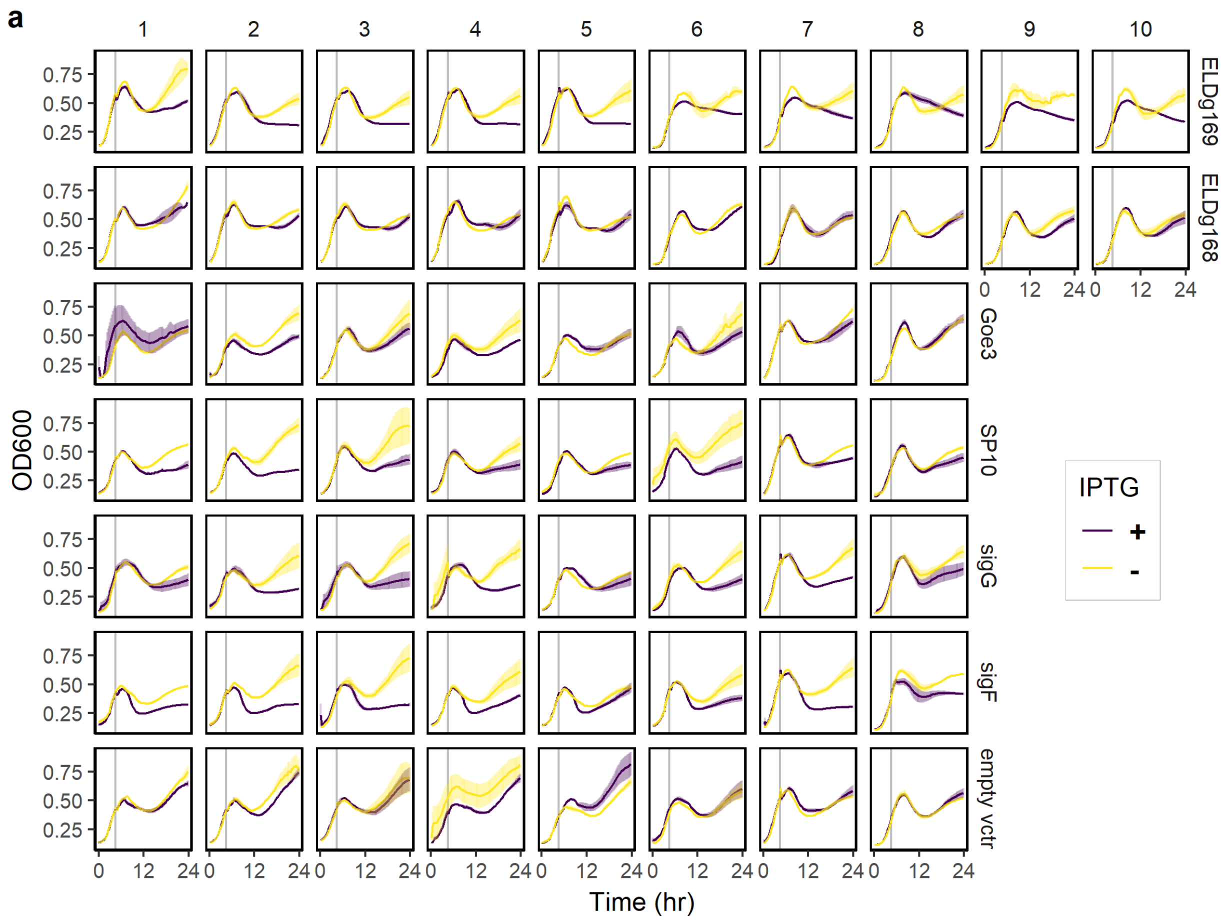

Fig. S4. *Continued on next page*

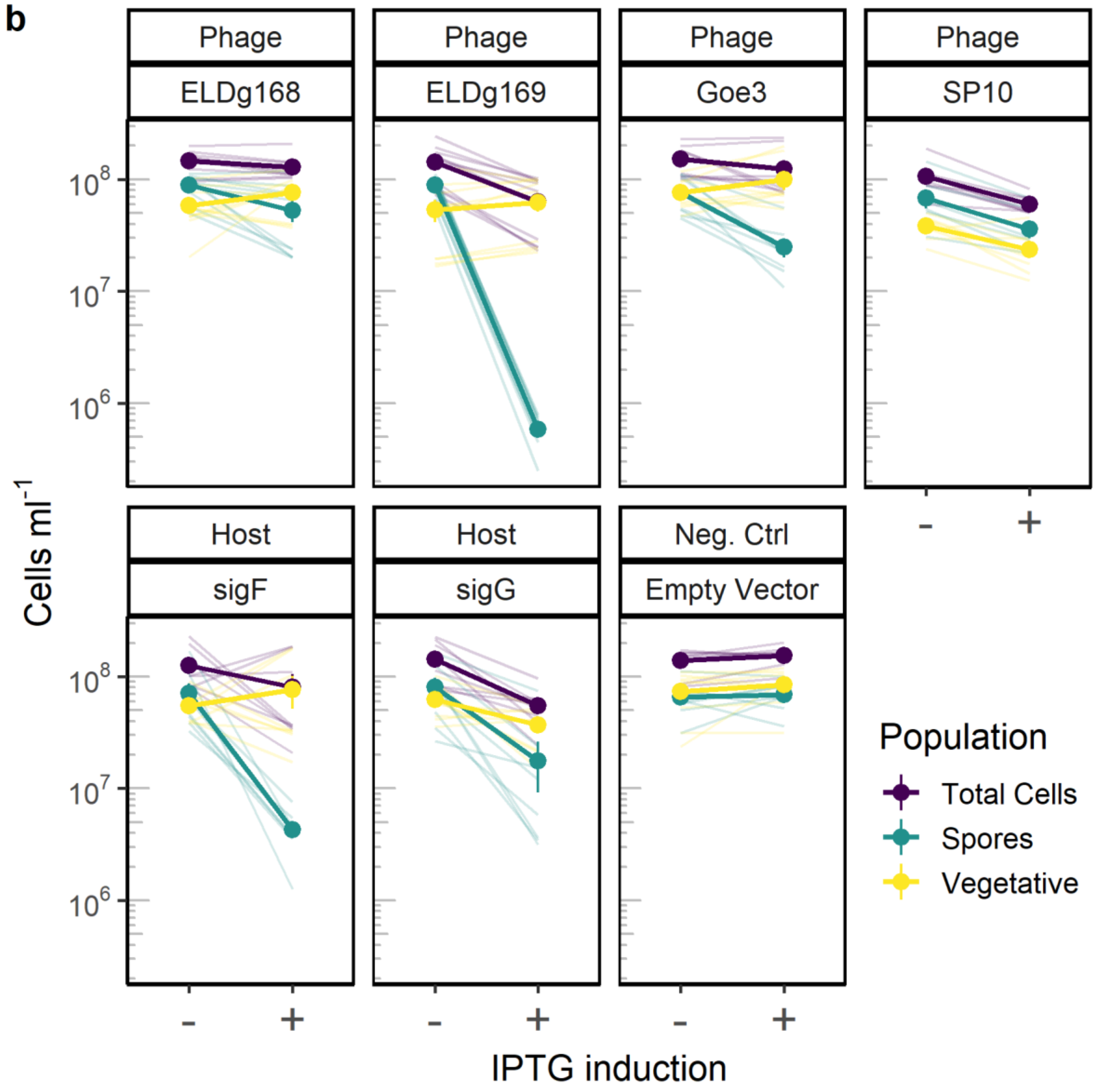

Fig. S4. *Bacillus subtilis* densities in sporulation assays. Sporulation was measured in strains with phage-derived or bacteria-derived (host) sigma factors under an IPTG- inducible promoter. The empty-vector negative control had the IPTG-inducible promoter without any sigma factor. **a,** Growth of *B. subtilis* during sporulation assays. Each panel shows the growth (mean ± SEM, n = 3) measured as optical density (OD600) over time of cultures with cloned sigma factor (row label on the right). Column numbers represent independent colonies from which experimental cultures were inoculated. Cultures were grown for 4.5 h (grey vertical line) in sporulation media before IPTG was added to half of them. Samples for quantifying spores and vegetative cells by flow cytometry were taken at 24 h. **b,** Effect of sigma factor induction on endpoint cell density. Cells were quantified and classified by flow cytometry after 24 h (spores + vegetative = total cells). Points connected by thick lines represent mean ± SEM of independent colonies (n ≥ 8). Thin lines connect values of each induced clone and its non-induced paired control.
