# Supplementary material for: Phage-Encoded Sigma Factors Alter Bacterial Dormancy
Source: mSphere. 2022 Jul 20;7(4):e00297-22. doi: 10.1128/msphere.00297-22 (PMC9429907; doi:10.1128/msphere.00297-22)
Supplement: TABLE S2 [file msphere.00297-22-s0008.pdf]

Table S2. Statistical results for sporulation of cells expressing cloned sigma factors.

Table S2a. Spore yield statistics. We used flow cytometry to quantify spores and vegetative cells. With this data, we calculated the fraction of spores in cultures of *Bacillus subtilis* strains with sigma factors cloned under an IPTG-inducible promoter (indicated in “group 1” column), or with empty vector controls (“group 2”). The mean value of each group is the ratio of spore fraction in induced vs. non-induced cultures (n > 8). We used a two-tailed Welch two Sample *t*-test, (difference in means ≠ 0) to compare cloned gene effect on sporulation between each of the cloned genes and the empty vector controls.

**Group 1**	**Group 2**	***t*-value**	**df**	***P*-value**	**adjusted *P*** [1]		**Mean group 1**	**Mean group 2**	**Difference in means ± CI** [2]
sigF	empty vector	6.432	8.199	0.000182	0.000545	***	0.1585	1.024	0.865 ± 0.309
sigG	empty vector	2.769	13.86	0.01519	0.02279	*	0.4906	1.024	0.533 ± 0.413
ELDg168	empty vector	2.097	15.12	0.05324	0.06389		0.6611	1.024	0.363 ± 0.368
ELDg169	empty vector	7.802	7.003	0.000107	0.000545	***	0.01658	1.024	1.01 ± 0.305
Goe3	empty vector	4.387	8.864	0.001818	0.003635	**	0.4203	1.024	0.603 ± 0.312
SP10	empty vector	0.4636	10.36	0.6525	0.6525		0.9567	1.024	0.067 ± 0.321

1. Multiple testing correction by the Benjamini, Hochberg, and Yekutieli method (* = $P < 0.05$, ** = $P < 0.01$, *** = $P < 0.001$)
2. CI = 95 % confidence interval

Table S2b. Statistics associated with total cell densities following sigma factor induction. We used flow cytometry to quantify spores and vegetative cells in *Bacillus subtilis* strains with cloned sigma factor genes. We used a two-tailed One Sample *t*-test (mean ≠ 0) to compare the difference in cell densities between induced and non-induced paired samples.

**Cloned gene**	**Cell type**	***t*-value**	**df**	***P*-value**	**Adjusted *P*** [1]		**Mean ± CI** [2]
empty vector	spores	0.3994	7	0.7015	0.7015		3.37e+06 ± 1.99e+07
sigF	spores	-4.05	7	0.00487	0.01136	*	-6.62e+07 ± 3.86e+07
sigG	spores	-5.491	7	0.0009151	0.005003	**	-6.28e+07 ± 2.70e+07
SP10	spores	-4.402	7	0.003148	0.009444	**	-3.18e+07 ± 1.71e+07
Goe3	spores	-5.453	7	0.000953	0.005003	**	-5.14e+07 ± 2.23e+07
ELDg168	spores	-4.114	9	0.002622	0.009444	**	-3.58e+07 ± 1.97e+07
ELDg169	spores	-13.81	9	2.30E-07	4.83E-06	***	-8.80e+07 ± 1.44e+07
empty vector	vegetative	1.115	7	0.3015	0.3333		1.10e+07 ± 2.33e+07
sigF	vegetative	0.7883	7	0.4564	0.4792		2.09e+07 ± 6.28e+07
sigG	vegetative	-2.341	7	0.05178	0.09062		-2.51e+07 ± 2.53e+07
SP10	vegetative	-2.981	7	0.0205	0.03913	*	-1.47e+07 ± 1.17e+07
Goe3	vegetative	1.569	7	0.1607	0.2249		2.35e+07 ± 3.54e+07
ELDg168	vegetative	1.768	9	0.1108	0.179		1.76e+07 ± 2.25e+07
ELDg169	vegetative	1.368	9	0.2045	0.2526		9.13e+06 ± 1.51e+07

Table S2b. continued

**Cloned gene**	**Cell type**	***t*-value**	**df**	***P*-value**	**Adjusted *P* [1]**		**Mean ± CI [2]**
empty vector	total cells	1.49	7	0.1797	0.2359		1.44e+07 ± 2.28e+07
sigF	total cells	-1.191	7	0.2723	0.3177		-4.52e+07 ± 8.97e+07
sigG	total cells	-4.109	7	0.004523	0.01136	*	-8.79e+07 ± 5.06e+07
SP10	total cells	-4.522	7	0.002727	0.009444	**	-4.66e+07 ± 2.44e+07
Goe3	total cells	-1.599	7	0.1539	0.2249		-2.79e+07 ± 4.12e+07
ELDg168	total cells	-2.896	9	0.0177	0.03718	*	-1.82e+07 ± 1.42e+07
ELDg169	total cells	-8.405	9	1.49E-05	0.0001563	***	-7.89e+07 ± 2.12e+07

1. Multiple testing correction by the Benjamini, Hochberg, and Yekutieli method (* = $P < 0.05$, ** = $P < 0.01$, *** = $P < 0.001$)
2. For each paired samples the difference in cell densities (per mL) are calculated as *induced culture – non-induced culture.* CI = 95 % confidence interval
