# Supplementary material for: Phage-Encoded Sigma Factors Alter Bacterial Dormancy
Source: mSphere. 2022 Jul 20;7(4):e00297-22. doi: 10.1128/msphere.00297-22 (PMC9429907; doi:10.1128/msphere.00297-22)
Supplement: FIG S5 [file msphere.00297-22-s0005.pdf]

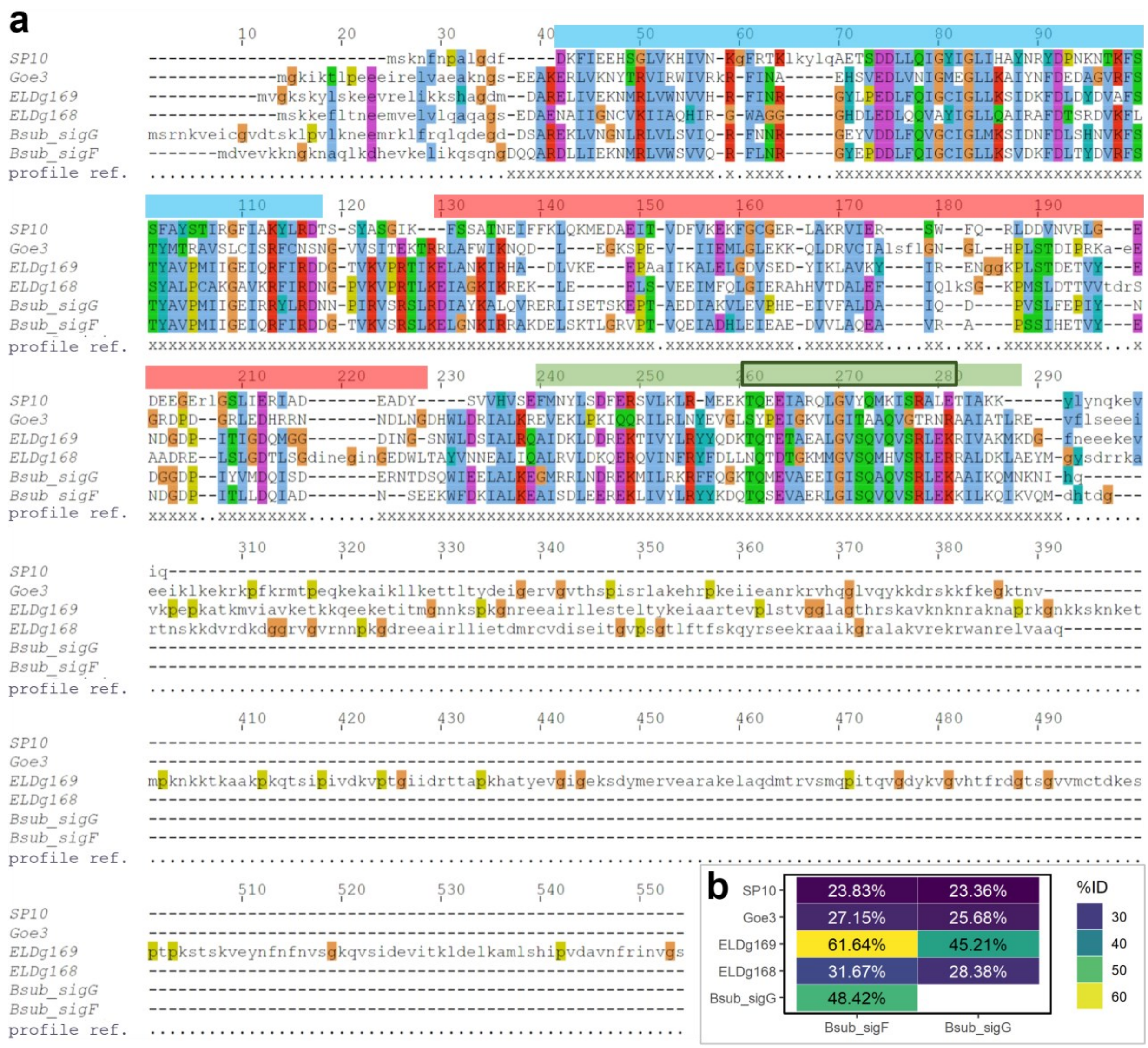

Fig. S5. *Continued on next page*

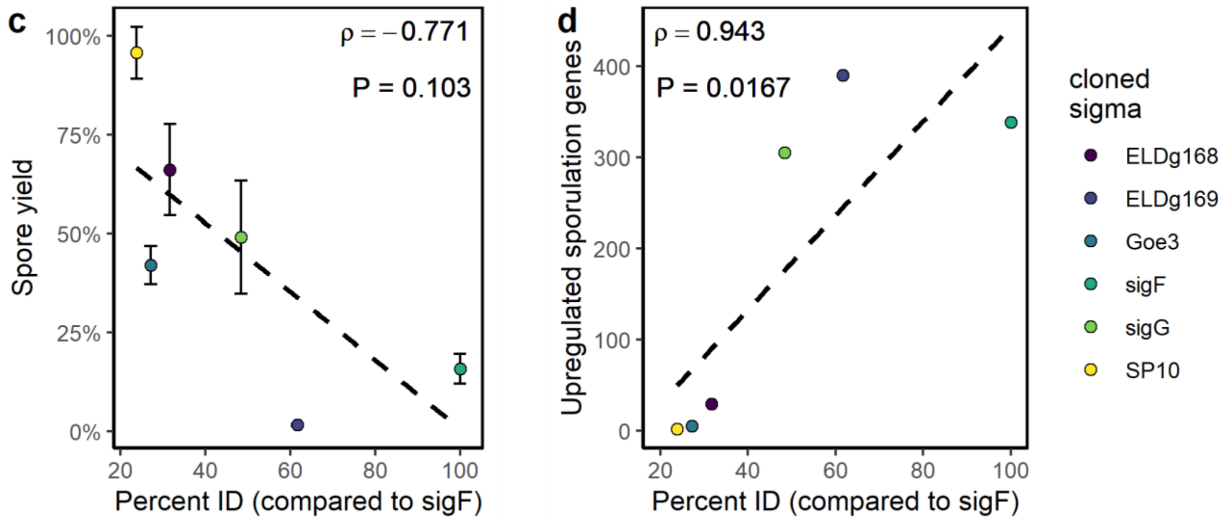

Fig. S5. Sporulation response corresponds with the identity of sigma factors induced in *Bacillus. subtilis*. **a**, Multiple sequence alignment of sigma factors cloned in this study. Protein sequences from phages and from *Bacillus subtilis* (Bsub) were aligned to the protein profile of the RNA polymerase sigma-70 factor, sigma-B/F/G subfamily (TIGR02980) using hmmalign (HMMER v3.3). The profile reference positions are marked with 'x' at the bottom of the alignment. Functional sequence regions of the host sigma factors are depicted above the alignment by color bars: region 2 in blue, region 3 in red, and region 4 in green, with the helix-turn-helix motif marked by a black box. Functional region annotation from SWISS-MODEL data for *B. subtilis* genes *sigF* (P07860) and *sigG* (P19940). **b**, Protein identities from the alignment. The identities were calculated using esl-alipid (part of the Easel library of HMMER v3.3) after trimming the N and C termini beyond the profile reference positions (using hmmalign –trim). **c-d**, The amino acid identity between cloned sigma factors and *B. subtilis sigF* is negatively correlated with the mean spore yield (**c**) (see Fig. 4) when induced during sporulation and positively corelated with the number of upregulated sporulation genes (**d**) when induced in exponential phase (see Fig. 3). Spearman's rho (ρ) and the associated *P*-value (*P*), and linear model (dashed line) were calculated in R. Error bars in **c** represent SEM.
