# Supplementary material for: Phage-Encoded Sigma Factors Alter Bacterial Dormancy
Source: mSphere. 2022 Jul 20;7(4):e00297-22. doi: 10.1128/msphere.00297-22 (PMC9429907; doi:10.1128/msphere.00297-22)
Supplement: TABLE S3 [file msphere.00297-22-s0009.pdf]

Table S3. Sigma factor protein groups of homologs

Table S3a. Viral orthologous groups (VOG) of sigma factors*

Group Name	Consensus Functional Description	Protein Count	Species Count
VOG00050	sp|P33658|RPSG_CLOAB RNA polymerase sigma-G factor	315	238
VOG01445	REFSEQ sigma-70 family RNA polymerase sigma factor	18	18
VOG02363	sp|P04524|SIGML_BPT4 RNA polymerase sigma-like factor	372	371
VOG10659	REFSEQ putative sigma factor	5	5
VOG12686	sp|P03048|RP28_BPSP1 RNA polymerase sigma GP28 factor	6	6
VOG13572	REFSEQ RNA polymerase sigma factor	4	4
VOG19786	REFSEQ sigma70, RNA polymerase sigma factor, positive control factor Xpf	2	2
VOG22313	REFSEQ sigma-70 family RNA polymerase sigma factor	2	2
VOG28223	REFSEQ RNA polymerase sigma factor SigF	2	2

*Data from VOG release vog209, vogdb.org

Table S3b. TIGRFAM protein families of bacterial sigma factors*

ID	Accession	Description	Label in main Fig. 3
rpoH_proteo	TIGR02392	alternative sigma factor RpoH	other
RpoD_Cterm	TIGR02393	RNA polymerase sigma factor RpoD	other
rpoS_proteo	TIGR02394	RNA polymerase sigma factor RpoS	other
rpoN_sigma	TIGR02395	RNA polymerase sigma-54 factor	other
FliA_WhiG	TIGR02479	RNA polymerase sigma factor, FliA/WhiG family	other
spore_sigmaE	TIGR02835	RNA polymerase sigma-E factor	sigE
spore_sigmaK	TIGR02846	RNA polymerase sigma-K factor	sigK
spore_sigG	TIGR02850	RNA polymerase sigma-G factor	sigG
spore_sigH	TIGR02859	RNA polymerase sigma-H factor	other
spore_sigF	TIGR02885	RNA polymerase sigma-F factor	sigF
spore_sigI	TIGR02895	RNA polymerase sigma-I factor	other
sigma70-ECF	TIGR02937	RNA polymerase sigma factor, sigma-70 family	other
RpoE_Sigma70	TIGR02939	RNA polymerase sigma factor RpoE	other
Sigma_B	TIGR02941	RNA polymerase sigma-B factor	other
Sig70_famx1	TIGR02943	RNA polymerase sigma-70 factor, TIGR02943 family	other
SigH_actino	TIGR02947	RNA polymerase sigma-70 factor, TIGR02947 family	other
SigW_bacill	TIGR02948	RNA polymerase sigma-W factor	other
SigM_subfam	TIGR02950	RNA polymerase sigma factor, SigM family	other

Continued on next page

* Data from ncbi.nlm.nih.gov/hmm/TIGRFAMs/release_15.0

Table S3b. continued

ID	Accession	Description	Label in main Fig. 2
Sig70_famx2	TIGR02952	RNA polymerase sigma-70 factor, TIGR02952 family	other
Sig70_famx3	TIGR02954	RNA polymerase sigma-70 factor, TIGR02954 family	other
SigX4	TIGR02957	RNA polymerase sigma-70 factor, TIGR02957 family	other
SigZ	TIGR02959	RNA polymerase sigma factor, SigZ family	other
SigX5	TIGR02960	RNA polymerase sigma-70 factor, TIGR02960 family	other
SigBFG	TIGR02980	RNA polymerase sigma-70 factor, sigma-B/F/G subfamily	other
SigE-fam_strep	TIGR02983	RNA polymerase sigma-70 factor, sigma-E family	other
Sig-70_plancto1	TIGR02984	RNA polymerase sigma-70 factor, Planctomycetaceae-specific subfamily 1	other
Sig70_bacteroi1	TIGR02985	RNA polymerase sigma-70 factor, Bacteroides expansion family 1	other
Sig-70_gvs1	TIGR02989	RNA polymerase sigma-70 factor, Rhodopirellula/Verrucomicrobium family	other
Sig70-cyanoRpoD	TIGR02997	RNA polymerase sigma factor, cyanobacterial RpoD-like family	other
Sig-70_X6	TIGR02999	RNA polymerase sigma factor, TIGR02999 family	other
Sig-70_gmx1	TIGR03001	RNA polymerase sigma-70 factor, Myxococcales family 1	other
