# Supplementary material for: Phage-Encoded Sigma Factors Alter Bacterial Dormancy
Source: mSphere. 2022 Jul 20;7(4):e00297-22. doi: 10.1128/msphere.00297-22 (PMC9429907; doi:10.1128/msphere.00297-22)
Supplement: TABLE S4 [file msphere.00297-22-s0010.docx]

**Table S4.** List of strains, plasmids and primers used in this study

*Table S4a. Bacteria and phage strains*

|  | Strain | Source |
| --- | --- | --- |
| Bacteria | *Bacillus subtilis* Δ6 | Bacillus Genetic Stock Center. BGSCID 1A1299 ^1^ |
|  | *Bacillus subtilis* TS01 | Robert Hertel ^2^ |
|  | *Priestia megaterium* KM (Eldridge host) | Center for phage technology, Texas A&M University,  ATCC #13632 |
| Phage | SP10 | Félix d'Hérelle Reference Center for bacterial viruses of the Université Laval ^3^ |
|  | Goe3 | Robert Hertel ^4^ |
|  | Eldridge | Louise Temple ^5^ |

1. Westers H, Dorenbos R, Van Dijl JM, Kabel J, Flanagan T, Devine KM, Jude F, Séror SJ, Beekman AC, Darmon E. 2003. Genome engineering reveals large dispensable regions in *Bacillus subtilis*. Mol Biol Evol 20:2076-2090.
2. Schilling T, Dietrich S, Hoppert M, Hertel R. 2018. A CRISPR-Cas9-based toolkit for fast and precise in vivo genetic engineering of *Bacillus subtilis* phages. Viruses 10:241.
3. Yee LM, Matsumoto T, Yano K, Matsuoka S, Sadaie Y, Yoshikawa H, Asai K. 2011. The genome of *Bacillus subtilis* phage SP10: a comparative analysis with phage SPO1. Biosci Biotechnol Biochem 75:944-52.
4. Willms IM, Hoppert M, Hertel R. 2017. Characterization of *Bacillus subtilis* viruses vB_BsuM-Goe2 and vB_BsuM-Goe3. Viruses 9:146.
5. Reveille AM, Eldridge KA, Temple LM. 2016. Complete genome sequence of *Bacillus megaterium* bacteriophage Eldridge. Genome Announc 4: e0172815.

*Table S4b. Plasmids*

| **name** | **Backbone / RE digest** | **Insert amplicon / RE digest** | **source** |
| --- | --- | --- | --- |
| pJOE88889 | - | - | BGSC ECE358 |
| pSP10-sgRNA | pJOE88889 | SP10_KOsigF_1+2 / BsaI | This study |
| pSP10-delete120 | pSP10-sgRNA | SP10_KOsigF_3+4+5+6 / SfiI | This study |
| pGoe4-sgRNA | pJOE88889 | Goe3_KOsigF_1+2 / BsaI | This study |
| pDR110 | - | - | David Rudner,  Xindan Wang |
| pDAS1 | pDR110/NheI + SphI | oDAS1+oDAS2 /XbaI + SphI | This study |
| pDAS2 | pDR110/NheI + SphI | oDAS3+oDAS4 /XbaI + SphI | This study |
| pDAS3 | pDR110/NheI + SphI | oDAS5+oDAS5 /XbaI + SphI | This study |
| pDAS4 | pDR110/NheI + SphI | oDAS7+oDAS8 /XbaI + SphI | This study |
| pDAS5 | pDR110/NheI + HindIII | oDAS1+oDAS2 /XbaI + HindIII | This study |
| pDAS6 | pDR110/NheI + HindIII | oDAS1+oDAS2 /XbaI + HindIII | This study |

*Table S4c. Primers*

| **Name** | **target** | **Sequence*** |
| --- | --- | --- |
| SP10_KOsigF_1 | SP10 (sgRNA) | tacgGATCATACCTGTTGTAAGCG |
| SP10_KOsigF_2 | SP10 (sgRNA) | aaacCGCTTACAACAGGTATGATC |
| SP10_KOsigF_3 | SP10 (g120 flank) | taggatccggccaacgaggccCAAAAAGTTGTAATCGGTATTGACTGGGG |
| SP10_KOsigF_4 | SP10 (g120 flank) | aagtagtccactaCATAACTAGACCTCCCGTTGTTTTCC |
| SP10_KOsigF_5 | SP10 (g120 flank) | aggtctagttatgTAGTGGACTACTTCAAAACCTACGAAGG |
| SP10_KOsigF_6 | SP10 (g120 flank) | taggatccggccttattggccCTCGACTTGACTGTTATCTTTAGAAACAATGC |
| SP10_valid_F | SP10 (g120 flank) | CCCTAAAACCTCCCGAGCTG |
| SP10_valid_R | SP10 (g120 flank) | GTCTTCCATGATACCGCCCC |
| Goe3_KOsigF_1 | Goe3 (sgRNA) | tacgAGGGGTGCAACCCGTTCCCT |
| Goe3_KOsigF_2 | Goe3 (sgRNA) | aaacAGGGAACGGGTTGCACCCCT |
| Goe3_KOsigF_3 | Goe3 (g157 flank) | taggatccggccaacgaggccGTTTAAAGACATAAATGACCCTCGAATCGA |
| Goe3_KOsigF_4 | Goe3 (g157 flank) | ggtttttagacattCATAATAGCCACCTCTTATATAATCACATTCTGT |
| Goe3_KOsigF_5 | Goe3 (g157 flank) | ggtggctattatgAATGTCTAAAAACCAAGATAAGTTTATTGTATGGAA |
| Goe3_KOsigF_6 | Goe3 (g157 flank) | taggatccggccttattggccGCTCTAAGCTCTTCTACTGCGCT |
| Goe3_KOsigF_1 | Goe3 (g157 flank) | tacgAGGGGTGCAACCCGTTCCCT |
| Goe3_KOsigF_2 | Goe3 (g157 flank) | aaacAGGGAACGGGTTGCACCCCT |
| oDAS1 | SP10-F | ctctctagaacataaggaggaactactATGTCTAAGAATTTTAATCCAGC |
| oDAS2 | SP10-R | ctcgcatgcCTATTGTATCACCTCTTTTTGG |
| oDAS3 | Goe3-F | ctctctagaacataaggaggaactactATGGGTAAGATTAAAACC |
| oDAS4 | Goe3-R | ctcgcatgcTTAGACATTGGTTTTACCCTCC |
| oDAS1 | SP10-F | ctctctagaacataaggaggaactactATGTCTAAGAATTTTAATCCAGC |
| oDAS5 | 168SigF-F | ctctctagaacataaggaggaactactATGGATGTGGAGGTTAAGAAAAACG |
| oDAS6 | 168SigF-R | ctcgcatgcCTAGCCATCCGTATGATCCATTTG |
| oDAS7 | 168SigG-F | ctctctagaacataaggaggaactactGTGTCGAGAAATAAAGTCGAAATC |
| oDAS8 | 168SigG-R | ctcgcatgcTTATTGATGAATATTTTTATTCATTTGTTTGATAG |
| oDAS9 | pDR110 (screen) | CGTACGATCTTTCAGCCG |
| oDAS10 | pDR110 (screen) | AGAACGTTGCTCGAGGG |
| oDAS15 | Eldridge169-F | ctcaagcttacataaggaggaactactGTGGTGGGTAAGAGCAAATATC |
| oDAS16 | Eldridge169-R | ctctctagaTTAGCTTCCCACGTTAATACGG |
| oDAS17 | Eldridge168-F | ctcaagcttacataaggaggaactactATGAGTAAAAAAGAATTCT |
| oDAS18 | Eldridge168-R | ctctctagaTTACTGAGCAGCTACTAG |

***** Target binding nucleotides are in uppercase letters and 5’ overhangs are in lower case letters. Added restriction sites are underlined.
