# Supplementary material for: Phage-Encoded Sigma Factors Alter Bacterial Dormancy
Source: mSphere. 2022 Jul 20;7(4):e00297-22. doi: 10.1128/msphere.00297-22 (PMC9429907; doi:10.1128/msphere.00297-22)
Supplement: FIG S6 [file msphere.00297-22-s0006.pdf]

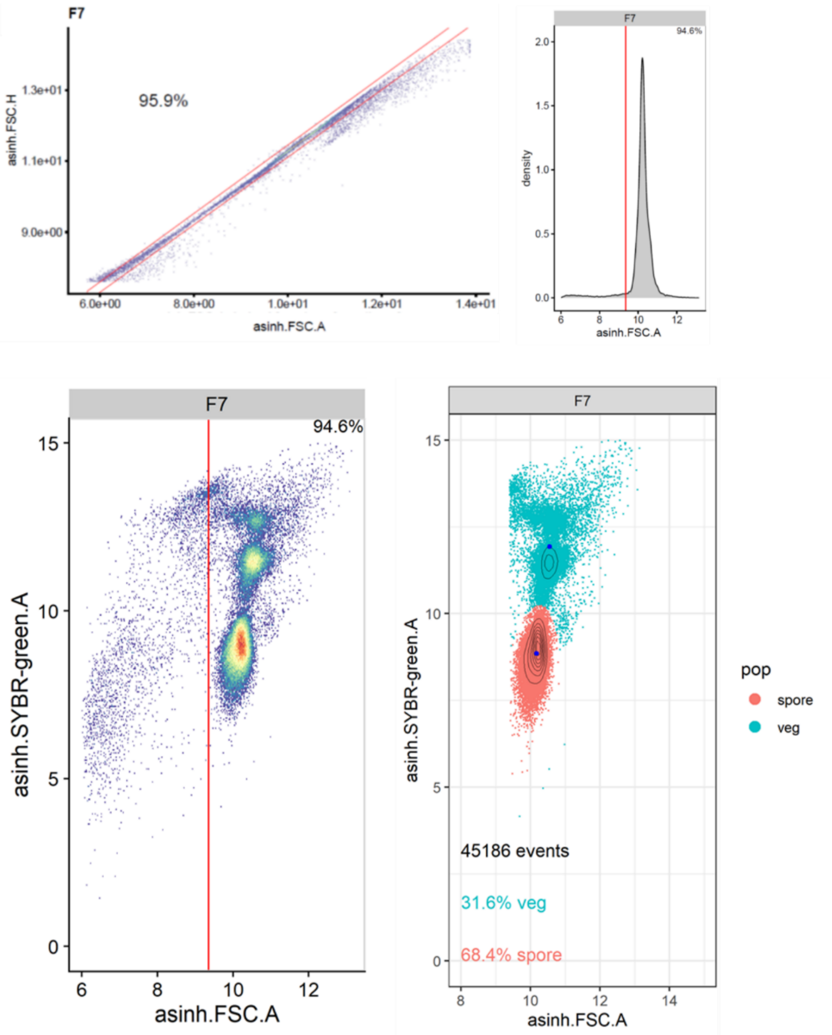

Fig. S6. Gating strategy used to quantify *Bacillus subtilis* spores and vegetative cells. **a-d**, Analysis was done on cells treated with DNA stain SYBR green that can penetrate vegetative cells, but not spores. Gating was done using an automated pipeline after the scattering and fluorescence signals were normalized by transformation with the hyperbolic arcsine ('asinh') function. **a**, Singlets were separated from doublets using the gate_singlet function of the flowStats R package applied to the transformed height ('h') and area ('a') of the forward scatter (FSC) signal intensity. **b**, Events attributed to noise (not cells) were identified as the lower tail of the distribution of transformed FSC area values in each sample using the rangeGate function of the flowStats package in R. **c**, noise gate applied to singlets. **d**, To cluster events into vegetative and spore populations we constructed a two population Gaussian mixture model using the non-induced control samples for each strain on each experimental run, using the no-IPTG control samples. The models were built using the Mclust function of the mclust R package based on the transformed data of the FSC and the SYBR green fluorescence intensity areas. The mean SYBR green intensity of the model populations (blue dots) was used to assign populations as vegetative (high SYBR) and spores (low SYBR). The model was then applied to classify events in all samples of the strain as spore and vegetative cells. Example sample is from experiment 4, showing a single culture (well F7) of the empty vector control strain, with no IPTG treatment.
